# Kinematics of Visually-Guided Eye Movements

**DOI:** 10.1371/journal.pone.0095234

**Published:** 2014-04-21

**Authors:** Bernhard J. M. Hess, Jakob S. Thomassen

**Affiliations:** Department of Neurology, University Hospital Zurich, Zurich, Switzerland; Scientific Institute Foundation Santa Lucia, Italy

## Abstract

One of the hallmarks of an eye movement that follows Listing’s law is the half-angle rule that says that the angular velocity of the eye tilts by half the angle of eccentricity of the line of sight relative to primary eye position. Since all visually-guided eye movements in the regime of far viewing follow Listing’s law (with the head still and upright), the question about its origin is of considerable importance. Here, we provide theoretical and experimental evidence that Listing’s law results from a unique motor strategy that allows minimizing ocular torsion while smoothly tracking objects of interest along any path in visual space. The strategy consists in compounding conventional ocular rotations in meridian planes, that is in horizontal, vertical and oblique directions (which are all torsion-free) with small linear displacements of the eye in the frontal plane. Such compound rotation-displacements of the eye can explain the kinematic paradox that the fixation point may rotate in one plane while the eye rotates in other planes. Its unique signature is the half-angle law in the position domain, which means that the rotation plane of the eye tilts by half-the angle of gaze eccentricity. We show that this law does not readily generalize to the velocity domain of visually-guided eye movements because the angular eye velocity is the sum of two terms, one associated with rotations in meridian planes and one associated with displacements of the eye in the frontal plane. While the first term does not depend on eye position the second term does depend on eye position. We show that compounded rotation - displacements perfectly predict the average smooth kinematics of the eye during steady- state pursuit in both the position and velocity domain.

## Introduction

Tracking the motion of a small object across a structured visual world challenges the constancy of spatial orientation due to the visual consequences induced by the eye movements. Since visually-guided eye movements have an intricate eye-position dependent kinematics the optic flow induced by tracking eye movements depends in a complex way on the location and geometry of the target trajectory in the visual field. In the simplest case a distant object may move in a plane, which happens to include the observer’s line of sight to the fixated target. The brain may then compensate for the movement-induced optic flow by simple image translation [1]. However, in many other situations the movement-induced optic flow is likely to be highly nonlinear, making a simple image translation impractical. According to H. v. Helmholtz perceptual stability is achieved by an estimation process of the visual consequences based on efference copy signals that are derived from the motor commands to the eye muscles [2]. This suggestion presupposes that the brain can efficiently estimate the three-dimensional kinematic consequences of the motor commands that generate the desired tracking motion of the eye. Although far vision is two-dimensional it matters for keeping visuo-spatial orientation stable to be informed not only about the current gaze displacement but also about how much the peripheral retina rotates about the line of sight. Since up to date there is not enough information about the geometric relationship between motor commands and three-dimensional ocular kinematics during smooth tracking of an object of interest, our understanding of the interactions between retinal and extra retinal signals remains necessarily limited. A major goal of this study is to bridge this gap starting from basic motor principles.

**Figure 1:**
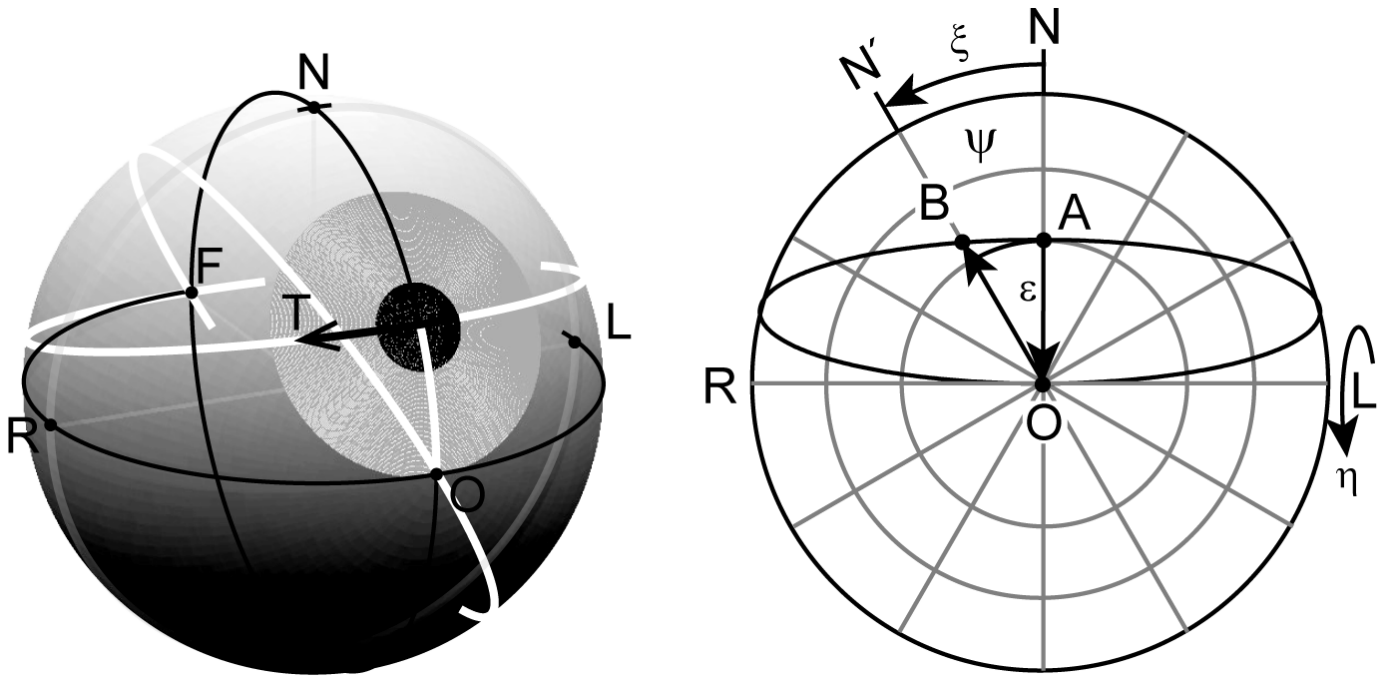
Virtual rotations required to approximate a smooth Listing-motion. To track a target that happens to move along a direction-circle (T: left panel), eye position signals holding the eye in the required plane of rotation could theoretically be derived by the following procedure (Helmholtz 1867): To obtain a smooth motion from A to B along the associated direction-circle (white circle through pupil, T, and F), the following four virtual rotations must be compounded: A first rotation in the sagittal plane NAO through an angle η subtending the arc AO, abbreviated by 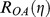, a second rotation in the frontal plane LNR through ξ subtending the arc NN′, abbreviated by 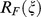, a third rotation in the meridian plane OBN′ through η′ subtending the arc OB, abbreviated by 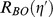, and finally a forth rotation in the eye′s coronal plane through –ξ, abbreviated 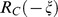 to eliminate the acquired torsion. Denoting by 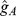 and 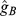 the unit gaze vectors parallel to 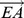 and 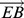, respectively, we have altogether 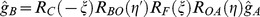. For small angles ξ, 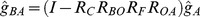 approximates a smooth Listing-motion from A to B along the direction-circle arc. Left panel, sketch of the eye: O, primary position; F, occipital position, antipodal to O; N, R, L, defining north, right and left directions in the eye’s coronal plane; direction- circle (white), circle passing through center of pupil and F. Right panel, front view onto the eye with spherical coordinate grid: ψ, meridian angle; ξ, rotation angle; ε, eccentricity relative to O along circles ψ = constant; η, rotation angle in planes ψ = constant.

There are two basic low level mechanisms that do constrain the kinematics of all visually-guided eye movements. One mechanism is Donders’ law, which asserts that the eye, while holding the head still, assumes always the same orientation for every fixation direction, independent of the preceding eye movement [3]. The other mechanism is Listing’s law, which implies that the eye can only assume certain specific orientations relative to the head [2], [4]–[6]. To reach those orientations the eye must rotate in planes that define, by way of intersection, a particular single direction in visual space, which has been called primary direction. This direction is distinguished by the unique property that any other direction in the visual field of fixations can be reached by a single rotation of the eye in the plane spanned by primary direction and the new desired direction. Despite its theoretical importance the notion of primary eye position and direction defies any more operational definition. Although there exist recursive procedures based on evaluating eye positions relative to a fixed reference position in far vision, while keeping the head upright and still [2], [4], [7], its neurophysiological significance in basic oculomotor research remains elusive. Since we rarely move the eyes with the head and body still the issue of how these basic mechanisms are imbedded in the larger context of head-free motor behavior has been intensively studied. While the head contributes to gaze movements, a major factor complicating the analysis of the basic role of Donders’ and Listing’s law in eye position control is the intricate interaction of visual and vestibular signals [8], [9]. Since the general relationship between eye position and position-dependent angular eye velocity signals related to Donders’ and Listing’s law is poorly understood, interactions with vestibular and other signals in the nested eye-head motor control system are difficult to discern. It appears therefore indispensable to more closely analyze this relationship in order to be able to segregate visual from vestibular and other effects in terms of the overall angular eye velocity in neurophysiological studies. In the current oculomotor literature it is often tacitly assumed that the angular velocity of a Listing-motion of the eye tilts by half the angle of gaze eccentricity with respect to straight ahead, although this is guaranteed only for fixed-axis rotations of the eye. The following analysis focuses on smooth tracking eye movements, which stand out as one of the prime examples of a Listing-motion [10]–[14]. Since visual targets can rarely be tracked by a single-axis rotation, it is still a mystery how such eye movements are generated within the constraints of Listing’s law. Here we propose a generic rotation algorithm based on the principle of minimizing ocular torsion. It generates smooth Listing-motions of the eye by operating linearly on the orientation of the line of sight for small rotation angles. Based on this algorithm we analyze the relationship between angular eye position and velocity of a general Listing-motion. Since such generic algorithm has not been known up to date, a Listing-motion of the eye has traditionally be conceived as a series of compounded rotations, also called virtual rotations to and from primary position between each fixation [2], [15] (Fig. 1). Taken as motor control strategy during smooth tracking movements such an algorithm not only implies a considerable amount of computation for every single instant of ocular motion but also lacks plausibility in terms of a time-critical strategy for target tracking. Besides combined eye-head gaze shifts smooth tracking movements with the head still or moving are typically non-fixed-axis rotations because the interesting target can rarely be smoothly tracked otherwise [16]–[18]. For testing the predictions of our mathematical analysis of the characteristics of a general Listing-motion, therefore we used three-dimensional eye movements that had been earlier recorded in non-human primates during linear and curvilinear smooth pursuit [11], [18].

## Results

### A Rotation Operator that Generates Listing-motions of the Eye

**Figure 2:**
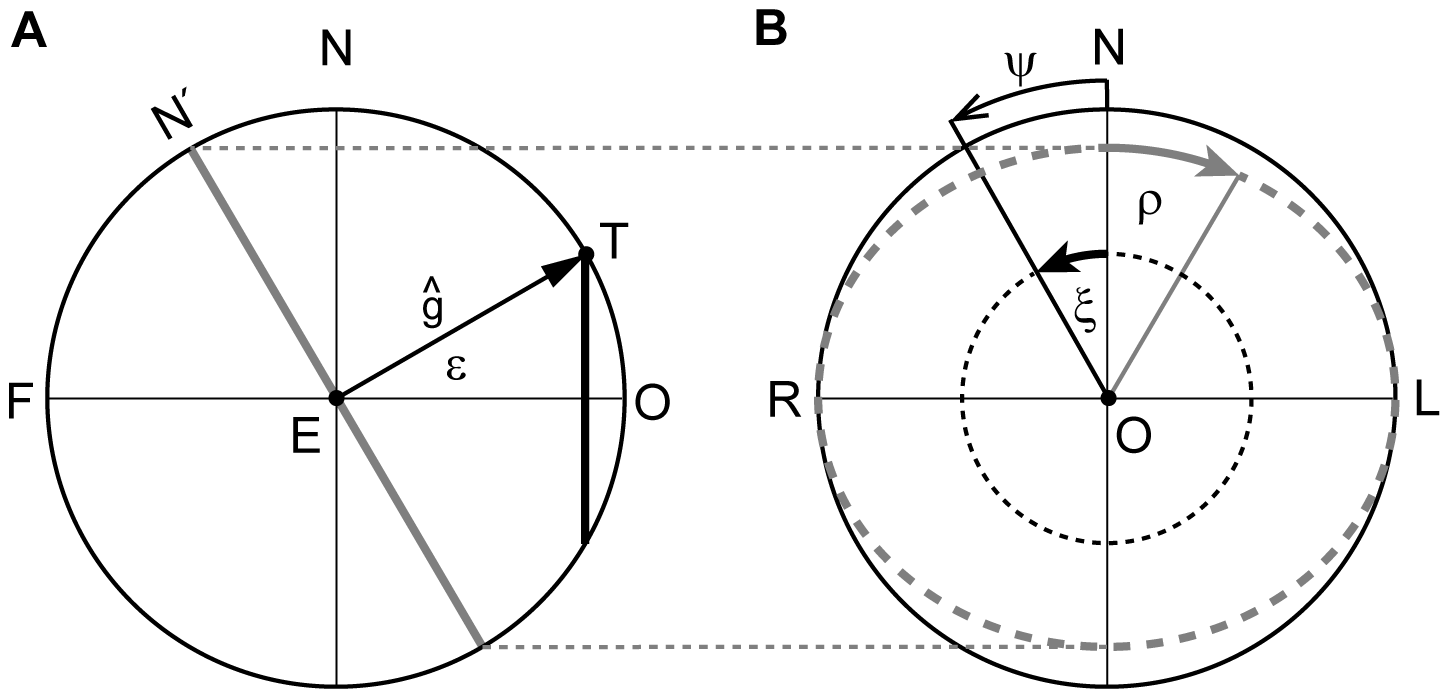
Geometry underlying the action of the operator R_CF_. (*A*) Side view on the spherical field of fixations, represented by a sphere with the eye (E) in the center, 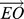 gaze direction straight ahead and F, occipital fixation point. The unit gaze vector 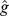 represents the direction of the line of sight, parallel to 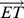, to the fixation point T. Fixation points are parameterized by the spherical polar coordinates ψ, ε. (*B*) Front view on the spherical field of fixations, displaying ξ, angle of ocular rotation in the frontal plane LNR; 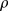, angle of ocular rotation in the eye’s coronal plane LN′R. For further details see text.

We show that it is possible to replace the virtual rotations illustrated in Fig. 1 by two explicitly defined single rotation operators. First we define a compound rotation operator 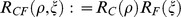 consisting of a first rotation of the eye through ξ in the head’s frontal plane followed by a rotation through 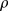 in the eye’s coronal plane and the requirement that the rotation angles fulfill the relation 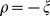 (Fig. 2). In contrast to compound rotations obtained by composing rotations in mutually orthogonal planes according to Euler, the rotation planes used to construct the compound rotation 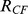 are not orthogonal to each other. In the following we show that this operator generates a Listing-motion by acting linearly on the line of sight for small rotation angles.

**Figure 3:**
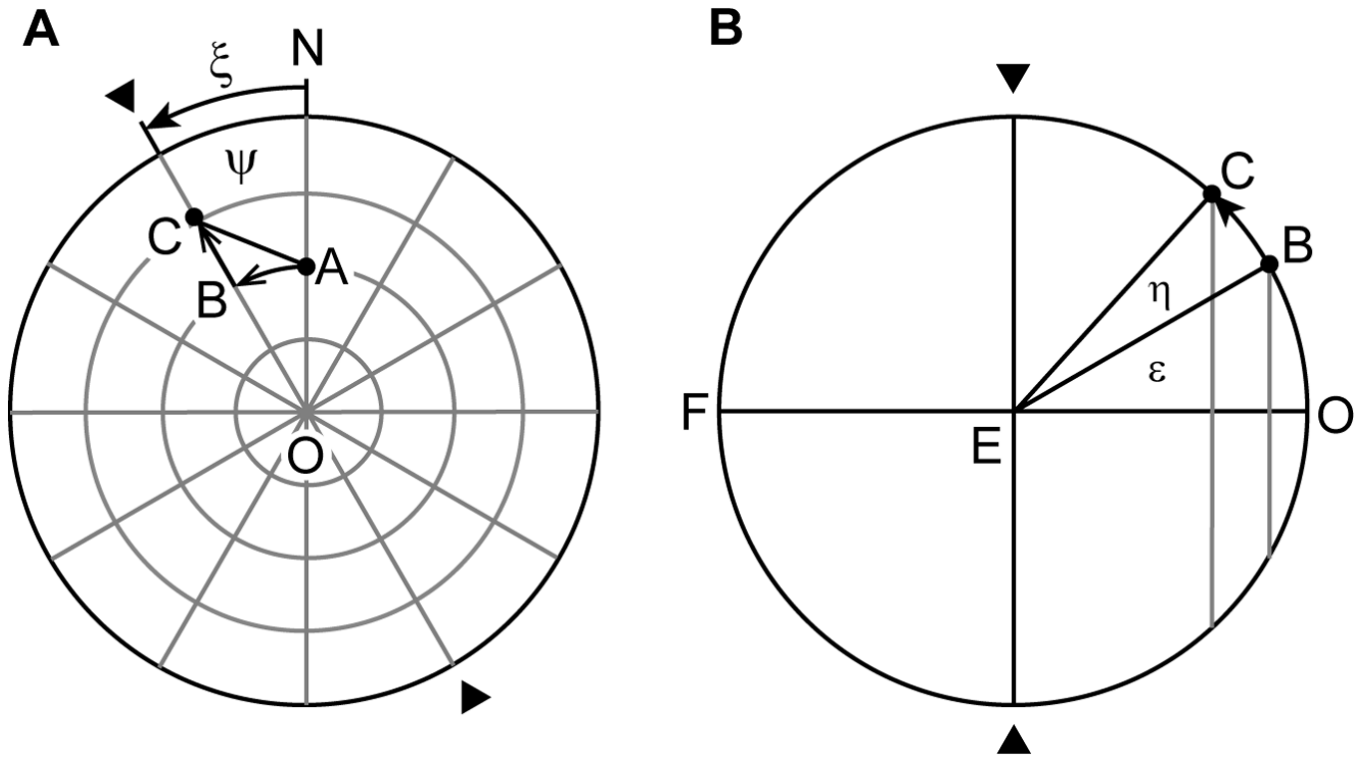
Approximation of a general Listing-motion in the spherical field of fixations. (*A*) Front view on the spherical field of fixations and (*B*) side view on the meridian plane through OBC. Under the action of the Donders-Listing operator 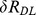 the fixation point A (gaze parallel to 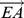) moves through a small angle ξ approximately along the arc AB from A to B. Similarly, under the action of the meridian operator 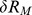 the new fixation point B (gaze parallel to 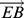) moves through a small angle η along the arc BC from B to C. If both the Donders-Listing and meridian operator act simultaneously the fixation point moves along the arc AC from A to C. Format similar as in Figures 1 and 2.



We define the direction of the line of sight by the unit gaze vector 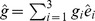 with coefficients 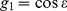, 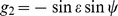 and 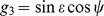 using the spherical polar coordinates ε and ψ (Figs. 1 and 2). The unit vectors 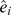 (i = 1, 2 and 3) represent a right-handed, head-fixed Cartesian coordinate system illustrated in Figs. 2 and 3 with 
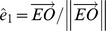
 pointing in direction straight ahead, 
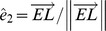
 pointing left and 
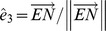
 pointing upward. We call the eye’s plane orthogonal to the line of sight the coronal plane in contrast to the frontal plane of the head, which is orthogonal to straight ahead (orthogonal to 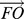 in Figs. 1, 2 and 3). To take advantage of the Clifford algebra of rotations, we introduce the basis vectors 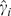 (i = 1, 2 and 3), which are defined by the properties 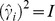 (identity) and 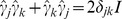 with 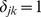 for j = k and 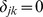 if j ≠k (for more details, see Text S1). In this basis, the unit gaze vector 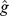 is represented by the 1-vector 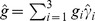, using the same coefficients g_i_ (i = 1, 2 and 3) as in Euclidean space. Furthermore, the frontal, sagittal, and horizontal planes are represented by the three 2-vectors 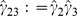, 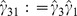 and 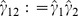, respectively. In the following we abbreviate linear combinations of these three basic 2-vectors by 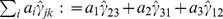. The coronal plane of the eye is represented by the 2-vector 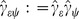 where 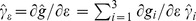 and 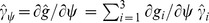. We now explicitly define the rotation operator 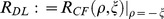 (Fig. 2):

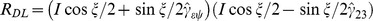





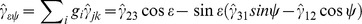
where ρ is the rotation angle in the eye’s coronal plane (given by 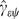), ξ is the rotation angle in the frontal plane (given by 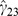) and ε is the eccentricity of the line of sight. In the direction straight ahead, we have 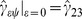 and thus 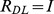. Note that the condition 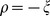 implies Donders’ law because it reduces the dimension of the manifold of ocular rotations from three to two. Since the torsion of visually-controlled eye movements is typically small we can expand the operator 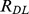 up to terms linear in ξ using the approximations 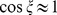 and 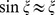. The resulting simplification renders the following calculations feasible and a posteriori proof to be sufficient for characterizing smooth visually-guided the eye movements. We will refer to the infinitesimal form of 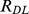 specifically as Donders-Listing operator 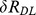:



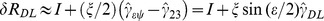





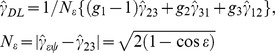



This linear operator functions as a generator of torsion-free rotations, i.e. motions that preserve Listing’s law in the oculomotor range up to second order corrections in ε and ξ (for a proof, see Text S2). In fact 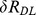 mediates a rotation of the line of sight in the head’s frontal plane 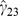 by rotating the eye in the tilted plane 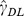. Indeed under the action of 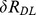 the gaze vector 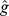 rotates from its current position through the angle ξ to the new position 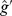 (see motion of unit gaze vector from 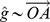 to 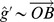 in Fig. 3A):
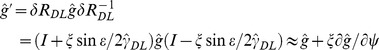



The approximation on the right side follows from the relation 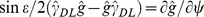 and by observing that 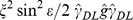 represents only a small contribution of second order. In contrast, the rotation plane of the eye ball tilts by half the line of sight’s eccentricity, namely through the angle 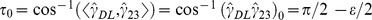. Indeed, the plane 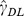 is approximately an eigenplane of the operator 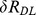: We have 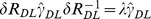 with eigenvalue 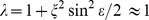 because 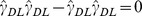 and 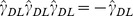 in the expansion of 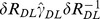. Thus the eigenvalue of 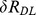 approximates the eigenvalue of a proper rotation up to corrections quadratic in ξ and ε/2, noting that ξ<1 and ε/2<1. Also note that at each gaze position the angle subtended by the rotation planes 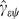 and 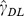 is 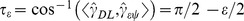, independent of the actual orientation of the eye.

To generate a general Listing-motion, the Donders-Listing operator must be combined with an operator mediating meridian rotations, that is horizontal, vertical and oblique rotations, whereby each of the two operators act in mutually orthogonal planes. The rotation plane of the meridian operator is defined by 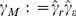 with 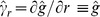 and 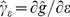. Thus we have:
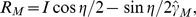





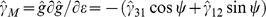



It can be approximated by 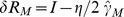 for small rotation angles 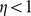. It moves the unit gaze vector along the meridian ψ = constant without changing the torsion of the eye. By compounding the meridian and Donders-Listing operator, eye positions can be generated that smoothly track arbitrary trajectories of objects of interest in visual space. Between each pair of transient fixation positions we have 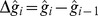
where.
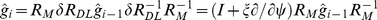
(2)moves the unit gaze vector from position 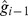 to position 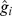. If the rotation in the meridian plane is small the rotation operator 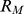 can be approximated by its linear version 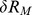. So far equation 1 and 2 suggest that essentially only two displacement signals, namely 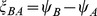 and 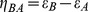 are needed to command a Listing-motion of the eye from position 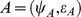 to 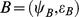. The two signals can be expressed in a one-to-one fashion in terms of azimuth and elevation of the eye as shown in the paragraph “Parameterizing Listing-motions in visual space”.

### The Total Angular Velocity of the Eye

Although recursive application of the infinitesimal compound rotation operator 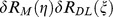 does generate eye movements in rotation planes that tilt by half the angle of gaze eccentricity, the question remains whether the angular velocity also follows the half-angle law of Helmholtz. To approach this question first we expressed the total rotation of the eye as 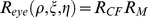 by compounding 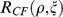 (as earlier defined) and the meridian rotation operator 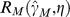. Left-multiplying the velocity d/d*t*(*R_eye_*) by the inverse 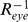, we obtained the angular velocity (see e.g. [19]):
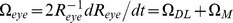
(3)with 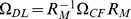, 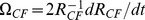 and 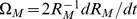. The expression for 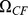 can be further broken down to 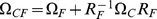 in terms of a roll angular velocity 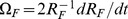 with 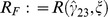 and a coronal or counter-roll angular velocity 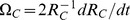 with 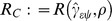. Clearly, the rotation plane of 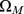 does not depend on the eccentricity of the line of sight in contrast to the term 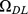. As a consequence, the total angular eye velocity does not obey the half-angle law of eye position (which always holds) because the rotation plane of 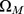 does not tilt. Next we analyzed the two terms on the right side of equation 3 in more detail.

### The Donders-Listing Angular Velocity

The counter-roll angular velocity is explicitly 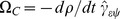. Similarly, the roll angular velocity is 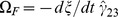. Substituted into 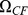 and evaluated at 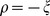, one obtains 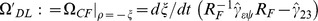. Because 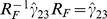, we have 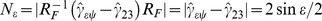, so finally we obtained:
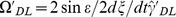





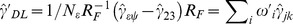





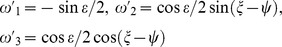



Thus, the plane of rotation of the angular velocity 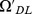 tilts in accord with the half-angle rule [4], at least if there is no meridian rotation involved. If there is such rotation, one has to go one step further by evaluating 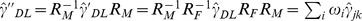, which yields the following expression, referred to as Donders-Listing angular velocity (for small rotation angles ξ):
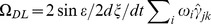
(4)




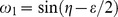





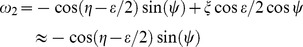





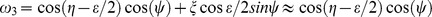



The angular velocity plane of the Donders-Listing angular velocity tilts through an angle 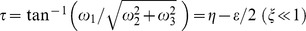, independent of the meridian ψ. Thus we found that the rotation plane of the Donders-Listing angular eye velocity must not coincide with the rotation plane of eye position. The half-angle law of eye position translates into a modified half-angle law of angular eye velocity. However, note that often the rotation angle η will be much smaller than ε/2, which obscures small differences between these two rotation planes.

### Meridian Angular Velocity

The contribution of 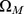
depends on the time rate of change of both the rotation angle η and the angular orientation ψ of the meridian plane. With the meridian plane 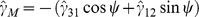 we have for the meridian angular velocity:
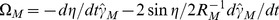



The second term in this relation implies a small change in ocular torsion because 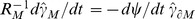 with 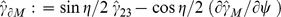. The two planes 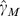 and 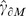 are mutually orthogonal. Note that 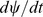 and 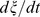 are equivalent. Thus we can write:
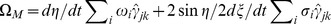
(5)




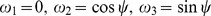





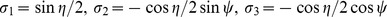



The dynamic interaction term on the right side of (5), which depends on the rotation angle η and the rotation velocity 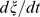 can make a significant contribution to ocular torsion as shown next.

### Ratio of Counter-roll to Target-induced Roll Angular Eye Velocity

To estimate the angular eye velocity induced by a visual target one has to know the counter-roll angular velocity, which contributes to the total angular velocity of the eye but not to the target angular velocity that is encoded by the fovea. We define the target-induced angular velocity as the difference 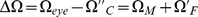 with the abbreviations 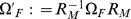 and 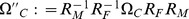. Dividing both sides by the magnitude 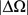 and rearranging the summands we obtained the following equation:
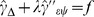



Here we have introduced the ratio 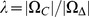, noting that 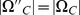 and the abbreviations 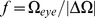, 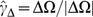 and 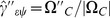. The λ-ratio is a complicated function describing the magnitude ratio of counter-roll to target-induced angular velocity in dependence of eye position (coordinates ε and ψ) and rotation (rotation angles ξ and η). To minimize accumulation of torsion, this ratio must be such that the counter-roll angular velocity compensates the target-induced angular velocity in the frontal plane (see Fig. 2B). Using this condition we evaluated the equation by computing the scalar product of *f* and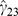, i.e. 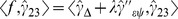, setting it equal to zero and solving for λ. We obtained for small rotation angles η (for calculation details, see Text S3):
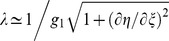
(6)


It describes the ratio of 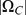 to 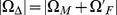 as a function of gaze eccentricity ε and the magnitude of the gradient 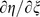, which we will refer to as counter-roll to roll angular velocity ratio. In case of a simple target-induced angular velocity in the frontal plane it reduces to 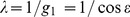, predicting that the counter-roll angular velocity must increasingly outmatch the roll angular velocity in magnitude as gaze eccentricity increases [18]. Conversely, it also predicts that the counter-roll angular velocity will undershoot the roll angular velocity in magnitude if there is a gradient 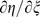 such that 
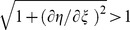
. In primary direction, one has 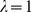, suggesting that fluctuations in roll angular velocity are matched on average in magnitude by counter-roll angular velocity signals.

### Parameterizing Listing-motions in Visual Space

**Figure 4:**
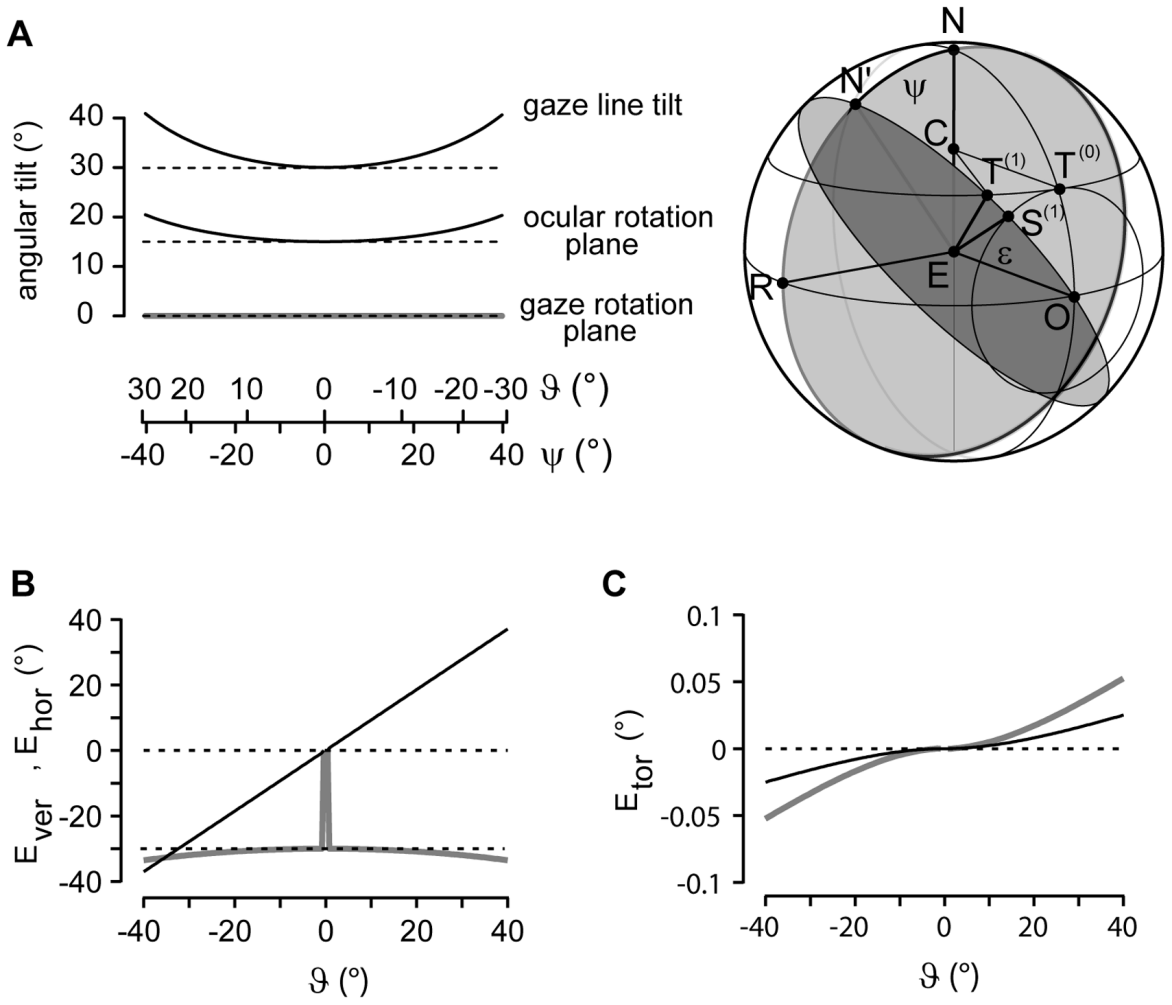
Minimizing ocular torsion during horizontal target tracking. *A, top black curve*: Angular tilt of the unit gaze vector 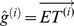 (i = 1, 2 …N) plotted against meridian angle ψ or azimuth angle 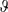. *Middle black curve*: Reconstructed eccentricity of ocular rotation plane plotted against meridian ψ or azimuth 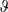. Note increasing tilt of these two curves with increasing absolute azimuth 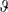. *Bottom thick gray curve*: Angular tilt of rotation plane of gaze line, remaining perfectly invariant during tracking motion. *B*: Simulated vertical (gray line) and horizontal component (black line) of 3D eye position plotted against azimuth (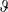); onset of tracking to the left and right at straight ahead. *C*: Simulated torsional component (black line: 10 Hz sampling rate as in *A* and *B*; gray line: 5 Hz sampling rate). Inset: azimuth 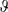 (not displayed), angle subtended by small circle arc from 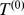 to 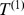; Listing’s plane, plane through E, N and R; line segment 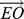, primary gaze direction; T^(0)^, initial target position; S^(1)^, intermediate position, virtually traversed while tracking the target from T^(0)^ to T^(1)^ (for more details see Results).

Target trajectories along straight lines in Euclidean space correspond to either small or great circle arcs in the spherical field of fixations. In contrast to great circles through the fixation point straight ahead, small circle -trajectories cannot be tracked by a torsion-free rotation of the eye in a single rotation plane. To approximate such trajectories, the eye must perform compounded rotations in meridian and frontal planes. The underlying transformations involve trigonometric relations between the azimuth and elevation of the desired fixation points and the respective meridian and eccentricity angles. For example, tracking of a target along an eccentric horizontal trajectory, corresponding to a horizontal circular arc in the upper hemisphere of the visual field, involves transformations of the unit gaze vector 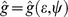 according to the relations.
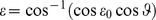





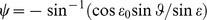
where 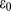 and 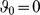 is the initial position of gaze after acquiring the target at position 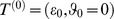 (Fig. 4, inset). Torsion-free tracking of this target can be achieved by repeatedly compounding Donders-Listing and meridian rotations to move the tip of the unit gaze vector first approximately along a small circular arc in the frontal plane, say from target position 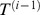 to 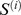 and then along the great circle arc through primary position and 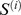 to the subsequent position 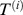 (as an example, see triangular path from 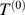 to 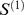 and to 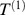 in Fig. 4). Since the resulting rotation is torsion-free up to second order corrections in ξ the overall torsion generated by a series of such compound rotations depends only on the chosen size of the Donders-Listing rotation steps. Simulations showed that the accumulated torsion across a ±40° horizontal excursion was linearly dependent on the number of sampling points (tested range 10≤ N ≤1000), reaching about 0.025° with an average 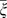 = 0.5° ±0.2° for N = 100 sampling points (Fig. 4). The angular ratio of the tilt of the rotation plane of the eye to the tilt of the gaze line followed the half-angle law [2] whereas the tilt of the rotation plane of the gaze line was virtually zero (Fig. 4A, B). Note also that the eccentricity of the gaze line increases significantly across the illustrated range of tracking compared to tracking onset at azimuth 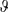 = 0 (compare the change of ε with 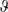 in Fig. 4A).

### Smooth Pursuit Angular Eye Position and Velocity Predicted from Target Motion

To check the predictions of equations 1 and 2 that two-dimensional position signals are sufficient to generate a Listing-motion, which is characterized by equations 3 to 6, we studied position and angular velocity of smooth pursuit eye movements during tracking of linear- and curvilinear-moving targets. For geometric reasons, such eye movements cannot be smooth and at the same time perfectly obey Listing’s law except in case of target-tracking along great circle arcs in visual space. Consider for example smooth tracking of a circularly moving object in a frontal plane, where the eye has to approximate the object’s path by a multi-sided polygon curve consisting of a series of small arcs of direction-circles. Indeed, such arcs only can approximate the circular path because of different curvatures: The curvature of a small circle arc with aperture 2ε (ε, gaze eccentricity) is always larger than the curvature of a direction-circle tangent to that arc (Fig. 1). Similar considerations hold for tracking objects along small circle arcs associated to straight lines in visual space (Fig. 4). Obviously, there must be a trade-off between smoothness of gaze motion and accord with Listing’s law during steady-state target tracking [18].

**Figure 5:**
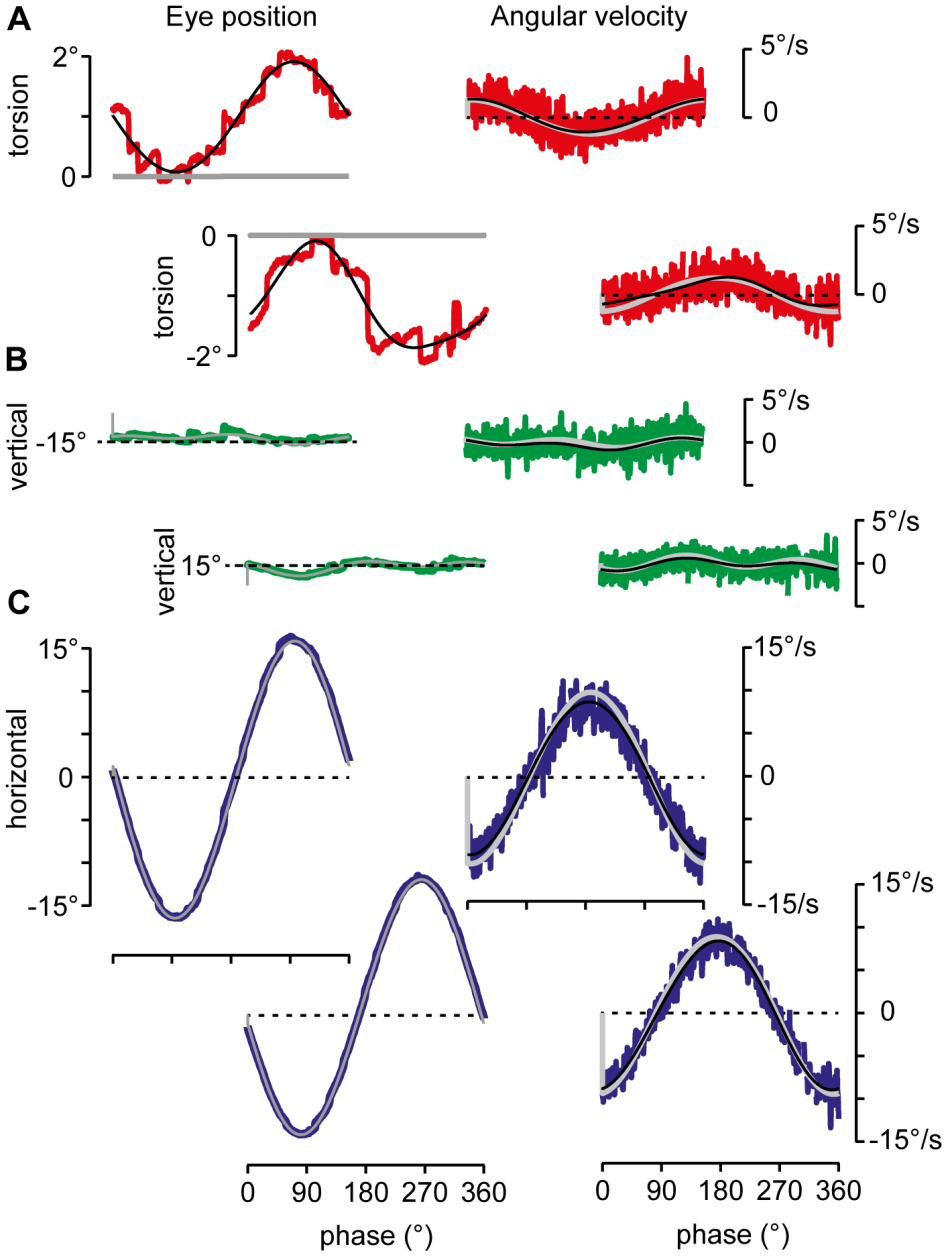
3D angular eye position and velocity reconstruction of horizontal tracking. *A to B, left two columns*: Reconstructed eye position (thick gray traces) during tracking at gaze 15° up and 15° down, superimposed on experimental 3D eye position in color (sinusoidal fits only shown for torsion). Note average torsional offset of about +1° and −1° for gaze up and down, respectively. Transformation to primary position would eliminate these offsets but not the saccadic modulation. *A to B, right two columns*: Reconstructed angular eye velocity (thick white traces) during tracking at gaze 15° up and 15° down, superimposed on experimental 3D angular eye velocity in color (sinusoidal fits, black traces). Torsional, vertical and horizontal experimental data in red, green and blue, respectively; for more details, see text.

Before addressing the complex paradigms of circular and elliptic tracking we present the results of linear smooth tracking, applying equations 1–5 to eye movement records obtained during tracking of targets that oscillated along horizontal or vertical small circles at various eccentricities relative to straight ahead. To reconstruct the ocular rotation, we used the initial orientation of the gaze line at tracking onset and an internal model estimating the target’s distance and orientation in the subject’s frontal plane (see Methods). From these pieces of information the rotation of the eye was reconstructed as a function of gaze orientation given by the polar angles ε and ψ (see Fig. 1 and 4). The reconstructed rotations reflected the smooth motion of the eye (up to first order in torsion, see equation 1), disregarding any step- like modulations of eye position due to minute saccades. Accordingly no modulation of torsional eye position was generated in contrast to the experimentally observed torsion due to saccades and subsequent drifts (Fig. 5A: compare sinusoidal fit in black with reconstruction in gray). The reconstruction predicts zero torsion offset relative to straight ahead, in agreement with the fact that the experimentally observed average torsion offset in the example illustrated in Fig. 5A added up to close to zero. Averaging across the five different gaze eccentricities and the two animals, the torsional modulation of eye position had an average amplitude (±SD) of 0.6° (±0.85°) and offset of 0.07° (±0.8°) during horizontal tracking (N = 44 cycles). Similarly, during vertical tracking the average amplitude (±SD) was 0.8° (±0.7°) with offset of 0.004° (±0.6°) (N = 47 cycles). Close inspection of vertical and horizontal eye positions revealed that the torsional saccades had also horizontal and vertical components during horizontal and vertical tracking, respectively, that changed direction at the turning points. The overall magnitude of these saccades was about 1° to 1.5°.

Again, the reconstructed eye position reproduced the average horizontal and vertical smooth modulation (Fig. 5C). Averaging across the five different eccentricities and the two animals, we found mean coefficients of determination (±SD) of vertical and horizontal reconstructions of 0.59±0.30 and 1.0±0.0002, respectively, in the horizontal tracking paradigm. The associated mean root-means-square (rms) errors (±SD) were 0.03±0.05 and 0.009±0.006 (N = 44). And similarly in the vertical tracking paradigm, the respective mean coefficients of determination were 1.0±0.0002 and 0.96±0.03 and mean rms-errors were 0.009±0.001 and 0.04±0.05 (N = 47).

According to equation (3), the experimental (slow phase) angular eye velocity should be the sum of Donders-Listing angular velocity (equation 4), the meridian angular velocity (equation 5) plus a significant contribution due to saccadic modulation of eye position and subsequent position drifts, which have been averaged out by the reconstruction of eye position. Whereas Donders-Listing angular velocity does tilt according to the half-angle law of Helmholtz the meridian angular eye velocity does not tilt (equation 5), except in cases where the gradient 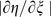 is large enough (equation 6). Averaging across the five different eccentricities and the two animals, we found mean coefficients of determination of the reconstruction-based torsional, vertical and horizontal angular velocity of 0.19±0.22, 0.10±0.07, and 0.96±0.01, respectively, with rms-errors of 0.88±0.12, 0.95±0.03, and 0.19±0.03 for horizontal tracking (N = 44). And similarly for vertical tracking we found mean coefficients of determination of 0.25±0.27, 0.90±0.04, and 0.25±0.16, respectively, with rms-errors of 0.81±0.15, 0.31±0.06, and 0.86±0.10 (N = 47).

**Figure 6:**
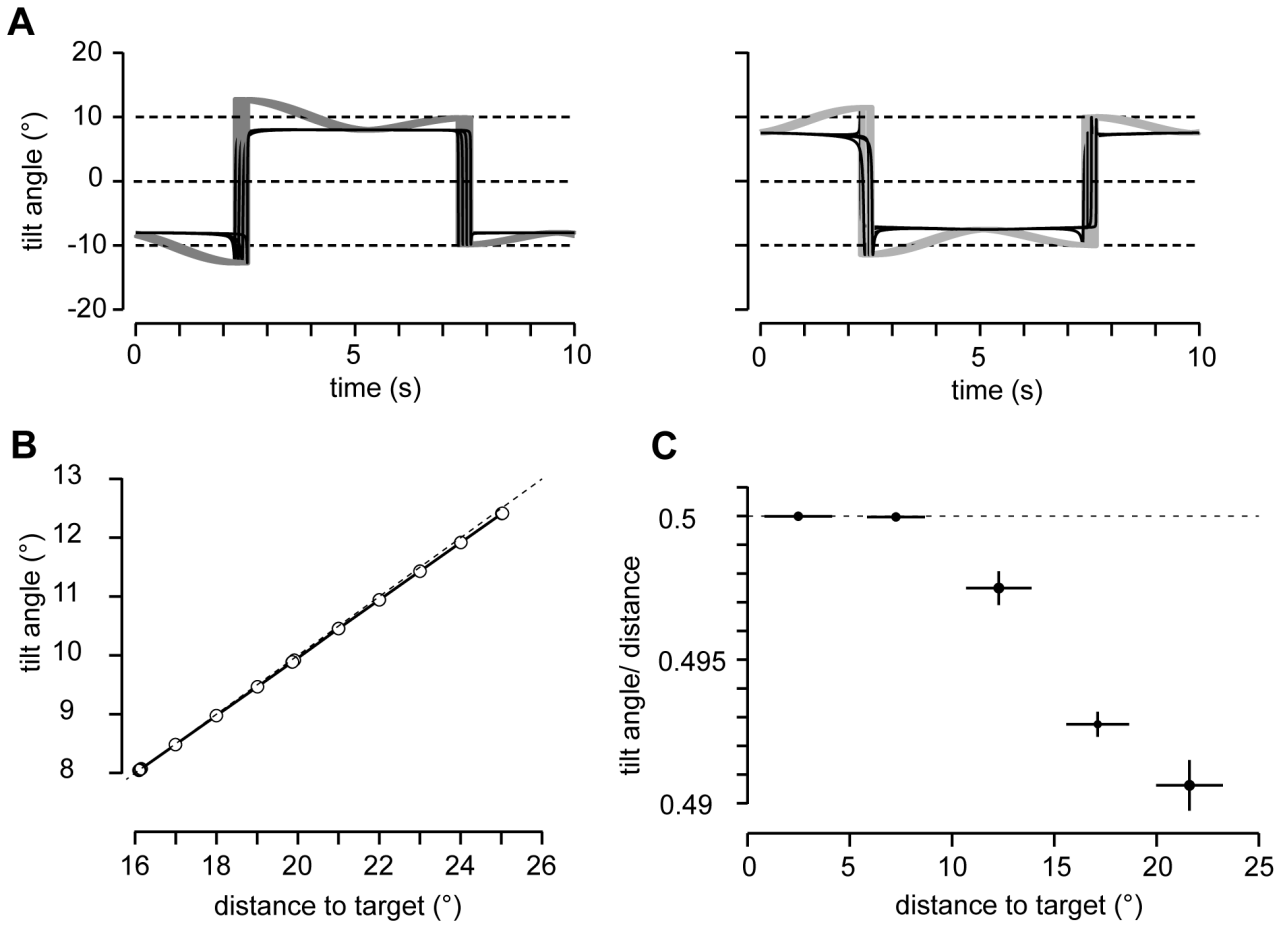
Tilt of angular eye velocity rotation planes during straight-line tracking. (*A*) Rotation plane tilt angles of total (Ω_eye_) and Donders-Listing (Ω_DL_) angular eye velocity during horizontal tracking in 15° gaze down, plotted across one oscillation cycle (5 response cycles superimposed, Ω_eye_: black traces, Ω_DL_: gray traces). (*B*) Rotation plane tilt of Donders-Listing angular velocity as a function of estimated target distance relative to straight ahead (data from 5 cycles). Note the small but steadily increasing deviation of tilt angles from the half-angle slope relation (dashed line) with increasing estimated target distance (least-squares fitted line through target positions: slope = 0.49, offset = 0.2°). (*C*) Average ratios of rotation plane tilt angles of Donders-Listing angular velocity to estimated target distance as a function of estimated target distance relative to straight ahead. Each of the data points was obtained by least-squares fitting tilt angles versus estimated target distance, collected in 5°-wide intervals across the range of 0° to 25°.

We also compared the degree of accordance with Listing’s law based on the experimental slow phase angular velocity and the total angular velocity derived from the reconstructed eye position. The conventional method compares the tilt of the angular eye velocity plane, calculated by 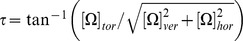 to the associated gaze eccentricity. Here **Ω** denotes the angular eye velocity vector obtained by least-squares sinusoidal fitting of slow phase angular velocity. The “associated gaze eccentricity” refers to the eccentricity of the point in the spherical field of fixations where the angular velocity rotation plane, the associated orthogonal meridian plane and the spherical field intersect. This point was estimated by averaging tilt angles and eye positions in the middle of each cycle between time t = 4 s to t = 6 s. On the other hand, we computed 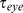 from the total eye angular velocity (equation 3) and 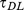 from the Donders-Listing angular velocity (equation 4) across each tracking cycle. Using the conventional approach we found tilt angle ratios of 0.51±0.03, 0.37±0.4, 0.09±0.06, 0.54±0.05 and 0.55±0.03 for the five fixation paradigms from gaze up to gaze down during horizontal tracking (N = 24 cycles, 1 subject). Using the reconstruction approach, the same animal showed a quite different picture. As predicted by equation 3, the tilt profile of the rotation plane of total angular velocity, 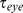, and that of Donders-Listing angular velocity, 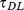 coincided only at the point in time where the eye (and the tracked target) crossed the vertical meridian during horizontal or the horizontal meridian during vertical tracking (Fig. 6A, compare black and gray traces). At these times during the tracking cycles, the instantaneous ocular rotation was tangential to current eye position. As the eye rotated away from these meridian crossing points during tracking, the tilt of the rotation plane of the total angular velocity remained constant (black traces in Fig. 6A), whereas that of Donders-Listing angular velocity increased in absolute terms due to the increasing distance relative to straight ahead (Fig. 6A, gray traces).

To further elaborate on this observation, we plotted the ratio of tilt angles to estimated target distances against estimated target distance. Target distance was defined by 
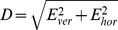
 (expressed in degrees). Tilt angles and target distances were determined at increments of one degree by averaging across ±0.25° throughout the tracking cycle. The tilt angles increased virtually linearly with slopes close to 0.50 as a function of target distance (Fig. 6B, example for horizontal pursuit at 15° gaze up). Averaging across the two subjects and paradigms we found that the ratio of tilt angle to target distance was 0.50 (±1×10^−5^, N = 290) in the position range of 5° to 10° around straight ahead and decreased thereafter to 0.4975 (±0.0006, N = 366) for distances between 10° and 15° and further to 0.492 (±0.001, N = 309) for distances beyond 15° (Fig. 6C).

### Smooth Tracking of Targets along Elliptic Trajectories

**Figure 7:**
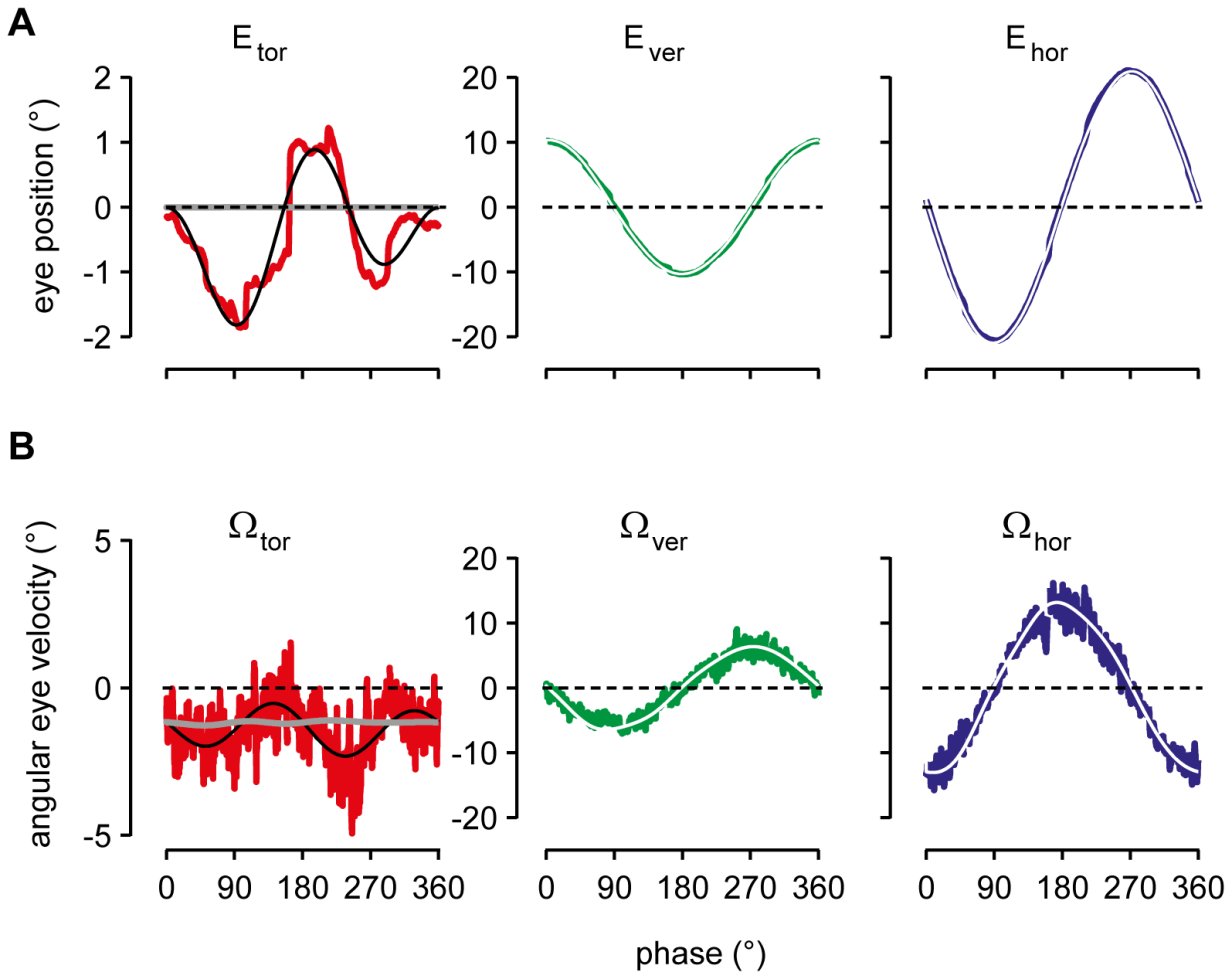
3D angular eye position and velocity reconstruction of elliptic tracking. (*A*) Reconstructed eye position superimposed on experimental 3D eye position in color (reconstructed torsion, thick gray trace; sinusoidal fit, black trace; reconstructed vertical and horizontal position, white traces; sinusoidal fits not shown). (*B*) Reconstructed angular eye velocity superimposed on experimental 3D angular eye velocity in color (reconstructed torsion, thick gray trace; sinusoidal fit, black trace; reconstructed vertical and horizontal angular velocity, white traces; sinusoidal fits not shown). Torsional, vertical and horizontal experimental data shown in red, green and blue, respectively; the minimal least-squares sinusoidal fits of vertical and horizontal eye position and angular eye velocity hardly differed from the reconstructed traces. For more details, see text.

As model for the following analysis served eye movement records of elliptic target trajectories with three different eccentricities (semi-major axis 20°, semi-minor axis 15°, 10° or 5°), oriented horizontally or vertically. Application of equations 1 and 2 based on the recorded meridian eye positions (angle ψ) and an internal model of elliptic motion (see Methods for details) perfectly reproduced the average smooth eye movement, except for the large saccadic modulation of torsional position (Fig. 7A). Averaged across the three classes of elliptic paradigms, we found a mean coefficient of determination of 1.0±0.001 for horizontal elliptic tracking with rms-errors of vertical and horizontal eye positions of 0.05±0.008 and 0.04±0.004, respectively (N = 155). Similar values were obtained for vertical elliptic tracking, yielding a mean coefficient of determination of 0.99±0.005 and average rms-errors of vertical and horizontal eye position of 0.05±0.008 and 0.04±0.004, respectively (N = 173).

The reconstructed eye position represented an average across the minute saccadic shifts in vertical and horizontal eye position. These small saccades had direction-specific torsional components, which often gave rise to torsional drifts in the amplitude range of 1–2° as shown earlier [18]. Accordingly, the reconstructed angular eye velocity did neither reproduce these experimentally observed torsional oscillations nor did it perfectly match the magnitude of the horizontal and vertical slow phase modulation. Averaged across the three elliptic paradigms, we found mean coefficients of determination for torsional, vertical and horizontal angular velocity of 0.07±0.03, 0.90±0.01, and 0.96±0.01, respectively, with rms-errors of 0.42±0.03, 0.17±0.02, and 0.10±0.01 for horizontal elliptic tracking (N = 155). And similarly mean coefficients of determination for torsional, vertical and horizontal angular velocity of 0.07±0.02, 0.91±0.02, and 0.95±0.01 respectively, with rms-errors of 0.35±0.02, 0.24±0.03, and 0.13±0.01 for vertical elliptic tracking (N = 173). According to the small torsional modulation in the position domain, the torsional angular eye velocity modulated in the range of about ±1°/s. Both these violations of Donders’ and Listing’s law in the position and velocity range averaged out across each response cycle (compare reconstructed and sinusoidal- fitted torsional angular velocity in Fig. 7A, B). To further corroborate this finding we compared the rotation planes.

**Figure 8:**
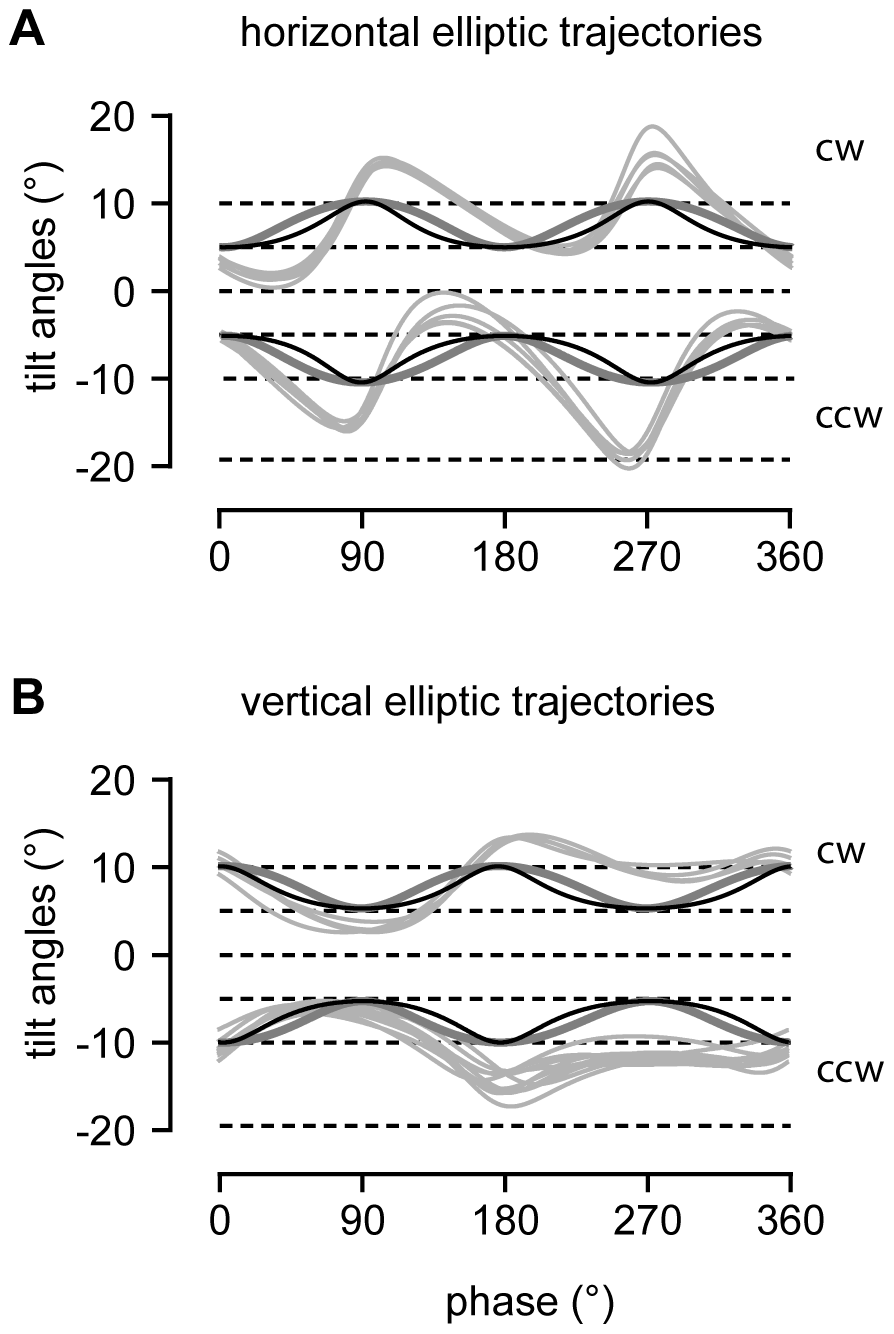
Tilting of angular eye velocity rotation planes during elliptic tracking. (*A*) Tracking of targets in clockwise (cw) and counterclockwise (ccw) direction along elliptic trajectories with major axes aligned with the head-horizontal and (*B*) head-vertical plane (ellipse major axis 20°, minor axis 10°). Traces illustrate tilt angles of rotation planes of reconstructed angular velocity (in black), Donders-Listing angular velocity (in dark gray) and sinusoidal-fitted angular velocity (in light gray). The disparity between reconstructed and Donders-Listing rotation planes is due to meridian rotations along the elliptic trajectory. The rotation planes of the sinusoidal-fitted angular velocities show large overshooting of the 5° and 10° levels predicted by the half-angle rule, particularly during tracking along horizontal elliptic trajectories; several single trials superimposed. Abscissa: phase 0°, up gaze; phase +90°, rightward gaze position (rotation sense of targets as seen from the subject).

We computed both the modulation of the tilt angle of the reconstructed angular eye velocity as well as that of the sinusoidal-fitted slow phase angular eye velocity and compared both of these to the tilt-modulation predicted by the Donders-Listing angular velocity. We found that in absolute terms the modulation of the tilt angle of the reconstructed angular eye velocity undershot the contour predicted by the gaze modulation during tracking in between the vertices of the elliptic track. This difference reflected the contribution of meridian angular velocity to the total angular velocity, which does not depend on eye position. In contrast, the modulation of the tilt angle of Donders-Listing angular velocity closely reproduced the expected contour: It modulated in synchrony with gaze eccentricity across the whole target cycle, as dictated by the elliptic trajectory (Fig. 8, compare black and dark-gray traces). On the other hand, the tilt angle of the sinusoidal fits of slow phase angular eye velocity (Fig. 8, light-gray traces), which included torsional saccadic drift velocities modulated in approximately the same range but phase shifted and distorted compared to both the Donders-Listing and the total angular eye velocity. Although this modulation greatly overshot the extreme vertices of the target trajectories, where the torsional angular velocities was high (≥50°/s) it conformed to the half-angle rule on average fairly well as documented in Table 1.

**Table 1:** Tilts of rotation planes of experimental and reconstructed angular eye velocity.

	Tracking	horizontal (N = 102)		vertical (N = 90)	
Ellipticeccentricity	Average gazeeccentricity × 1/2 (°)	Average experimentaltilt angle (°)	Average reconstructedtilt angle (°)	Average experimentaltilt angle (°)	Average reconstructedtilt angle (°)
e = 0.66	8.6	10.8±1.0	8.8±0.07	10.6±1.0	8.7±0.04
e = 0.87	6.9	8.8±0.8	7.1±0.06	8.7±1.1	7.0±0.07
e = 0.97	4.5	5.9±1.2	4.6±0.07	5.8±1.5	4.6±0.06

Average tilts (± SD) of rotation planes of experimental angular eye velocity, obtained from sinusoidal fits of slow phase angular eye velocity, follow approximately the half-angle rule. In contrast, average tilts (±SD) of rotation planes of reconstructed angular eye velocity based on equation 3 accorded with the half-angle rule in terms of estimated average gaze eccentricity. For a cycle per cycle comparison see Fig. 8.

### Ratio of Counter-roll to Roll Angular Velocity

**Figure 9:**
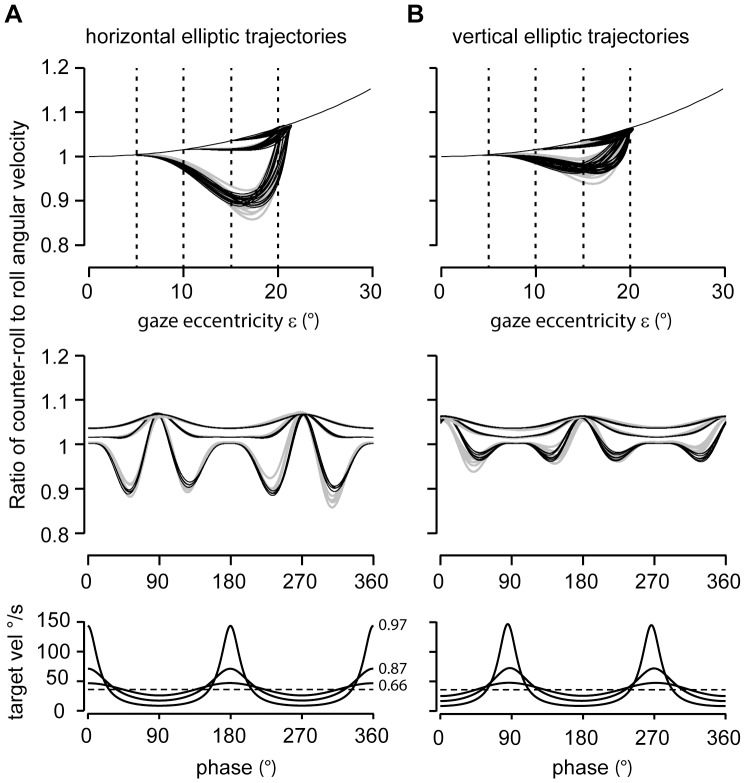
Ratio of counter-roll to roll angular velocity. Comparison of reconstructed versus experimentally estimated counter-roll to roll ratios during horizontal (*A*) and vertical (*B*) elliptic target tracking. *Upper panels*: Counter-roll to roll ratios obtained from single trials for three different elliptic eccentricities plotted against the angular eccentricity ε of the gaze line by superimposing reconstructed (black lines) on experimentally estimated data (gray lines). Dashed vertical lines indicate the extreme vertices of elliptic trajectories (e = 0.66, 0.87 and 0.97; a = 20°, semi-major axis, b = 15°, 10°, and 5°, semi-minor axes). The single black curve displays the curve 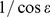, extending from 1 at ε = 0° to 1.15 at ε = 30°. *Middle panels*: Counter-roll to roll ratios as above plotted against tracking phase. Note increasing depth of modulation with increasing elliptic eccentricity, particularly during horizontal elliptic tracking. Abscissa: phase 0°, up gaze; phase +90°, rightward gaze position. *Bottom panels*: Torsional target velocity along the three elliptic trajectories with eccentricities 0.97, 0.87, and 0.66 plotted against phase angle. Dashed line indicates average torsional angular velocity across the three trajectories (36°/s).

We estimated the ratio of counter-roll to roll angular velocity using two independent procedures. First, we estimated this ratio from angular eye position and velocity records and found that it deviated from the expected 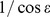 curve. Specifically, for tracking target trajectories with small and intermediate eccentricities (semi-minor axes b = 15°, 10° versus semi-major axis a = 20°), the ratio approximately stayed constant after the target crossed the vertex ε = b of the elliptic trajectory at values close to 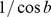 before turning towards and reaching the predicted value 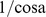 at the vertex ε = a (Fig. 9, light-gray traces in upper panels). For trajectories with large elliptic eccentricity (semi-minor axis 5°, semi-major axis 20°), it markedly undershot the curve 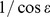 between the vertices at ε = a and ε = b. In each of these cases the ratio accorded with the values of 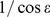 at the four vertices where the gradient 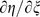 vanished, as predicted by equation (6) (Fig. 9, light-gray traces in upper panels). The same experimentally estimated counter-roll-to-roll ratios are also illustrated as a function of tracking phase (Fig. 9, light-gray traces in middle panels).

Secondly, we reconstructed the ratio of counter-roll to roll angular velocity based on equation 6 using the same set of polar angles ψ and ε as used for reconstructing eye position and angular eye velocity. We found that across all horizontal and vertical tracking paradigms the thus reconstructed ratio predicted the experimental ratio with an average coefficient of determination of 0.89±0.04 and average root-mean square error of 0.003±0.002 (N = 328, Fig. 9, black traces superimposed on light-gray traces in upper and middle panels).

## Discussion

We have shown that the rotation of the eye during general smooth tracking movements can be modeled by combining conventional rotations in horizontal, vertical or oblique planes with small displacements in the frontal plane. Further we have found that this novel type of compounded rotation - displacements can explain how the eye approximates rotations required to steer the fixation point along any desired path in visual space without accumulating torsion during smooth tracking. This rotation strategy of the eye has three characteristic features: First, it is the basis of Donders’ and Listing’s law. Second, it does not depend on where exactly primary position is located. Third, it relies on meridian rotations combined with linear displacements in the frontal plane, which explains the paradox of ocular kinematics that the fixation point can rotate in one plane while the eye rotates in other planes.

### A Generic Mechanism Underlying Donders’ and Listing’s Law

Since the early attempts of Helmholtz to derive Listing’s law from a visuo-motor error functional, which he called the principle of easiest orientation [2] (for a re-evaluation of Helmholtz’s approach see [15]), a number of other studies have shown that Listing’s law at least can be understood as a principle minimizing certain motor parameters [6], [15], [20]–[23]. The more recent discovery of fibro-muscular structures that alter the pulling directions of the oculomotor muscles has revived this discussion about the origin of Listing’s law by proposing that the so-called half-angle rule of angular eye velocity [4] is implemented by an ingenious neuro-mechanical mechanism located peripheral in the ocular plant [24]–[27]. This hypothesis hinges on the issue of how the rotational mechanics of the eye effectively works, particularly with regard to the mechanisms that underlie the half-angle law of eye positions in Listing’s law (for the half-angle law, see notion of direction-circle in [2]). Despite great progress, some of the basic visuo-motor mechanisms in oculo-motor control are still not well understood. For example, how can we look around a circle although Listing’s law forbids rotations of the eye in the frontal plane? Or why should primary position play such a pivotal role in Listing’s law when it is often found far from the center of the oculomotor range [4], [28]? The here proposed rotation-displacement mechanism solves these problems.

As to primary direction, assume that it is somewhere down from the center of the oculomotor range when looking straight ahead (with the head upright). Nonetheless the proposed displacement operator 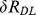 that controls rotations of the fixation point in frontal planes would not change the torsion of the eye with respect to straight ahead (up to corrections quadratic in the rotation angle). Similarly, any rotation in planes whose mutual intersections coincide with this direction would not change torsion either, even not in combination with the displacement operator 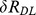. Taking primary position into account would thus not change the torsion of the eye. We conclude from this that, for maintaining visuo-spatial orientation constancy, it does not really matter where exactly primary eye position is located. However, it does make a difference in terms of computational load whether every single eye position had to be computed relative to primary position (see example in Fig. 1) or whether the proposed alternative rotation-displacement strategy is used.

Our approach also explains the paradox of different rotation planes of the line of sight and the eye ball. For example, under the sole action of the displacement operator 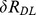, the fixation point can almost perfectly approximate a circular trajectory in a frontal plane while the underlying rotation of the eye occurs at any point in time in planes tilted by half the angle of gaze eccentricity relative to straight ahead. Indeed, these tilted planes are eigenplanes of torsion-free rotations of the eye up to quadratic corrections in the rotation angle ξ. Under the action of 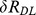, the fixation point displaces tangential to both its direction-circle, which is tilted by ε/2 and a frontal circle with opening angle ε relative to straight ahead. By adding up such displacements, the eye can generate an almost perfect circular trajectory of the fixation point while tracking the target. Similarly, during tracking of a target along a small circle arc, for example a horizontally moving target in the upper visual hemisphere, the rotation plane of the tracking fixation points remains almost perfectly parallel to the horizontal plane (Fig. 4). However, the eye actually rotates in planes that are tilted away from the horizontal plane by half the angle of gaze eccentricity, by combining small displacements in these tilted planes with rotations in meridian planes. Again the overall rotation is approximately torsion-free because these small displacements occur orthogonal to the meridian and tangential to direction-circle of the current fixation point at any point of time.

### The Half-angle Law of Eye Positions does not Readily Generalize to Angular Eye Velocity

Our reconstructions of 3D eye position perfectly matched 3D experimental eye positions except for the saccadic modulation of torsional eye position. Similarly, the minute saccadic displacements in both the vertical and horizontal eye position modulation were averaged out by this approximation. As expected, the thus reconstructed eye positions were in accord with the half-angle law up to angular corrections of the order of ξ^2^, where ξ is the rotation angle in the frontal plane (equations 1 and 2, Figs. 5A, 7A). The experimentally observed modulation of torsional eye position is due to the same saccades observed in horizontal and vertical eye position and the ensuing drifts. During straight-line tracking, the amplitudes of this modulation increased or decreased in a mirror-symmetric fashion relative to straight ahead, reaching about 1° at the most eccentric gaze position (Fig. 5A). The direction changed at the turning points during straight-line tracking and depended on the rotation-direction during circular or elliptic tracking eye movements [18]. The modulating saccades likely occur for geometric reasons. One of these reasons is to keep the target centered on the fovea. During straight-line tracking, a correction of eye position by 1° tangential to a maintained eccentric position of 15° generates an ocular torsion of about 0.13°. Repeated corrections in alternating direction during the tracking cycle can explain the observed modulation. Other reasons can be to correct and avoid accumulation of torsion. Since the Donders-Listing operator is a linear approximation it does not perfectly compensate the torsion associated with the generated eye movement. Without saccadic corrections, curvilinear smooth pursuit would first of all violate Donders’ law, which is of primary importance for visuo-spatial orientation constancy [18].

To provide a more formal argument, consider smooth tracking of a target that moves in a frontal plane along a circular path with radius 15° at 36°/s. With an assumed updating rate of eye position every 500 ms without any correction of ocular torsion, the proposed Donders-Listing mechanism would move the fixation point along an icosagon that approximates the target’s circular path. As a consequence, ocular torsion would accumulate by about 0.05° per smooth motion segment, increase to a maximum of about 0.5° after 10 segments (at about 180°) and decrease thereafter without hitting the initial zero position after 20 segments (at 360°): There would be a violation of Donders’ law amounting to about 0.5° at the end of one response cycle. This value would multiply by the number of continuously tracked cycles. In practice, however, subjects can easily track circular or even elliptic targets through several cycles without problem, although elliptic tracking is more challenging because of the potentially large torsional angular velocities.

For simplicity, we reconstructed 3D eye position at the same updating or sampling rate of 833.33 Hz as recording the experimental data. To check the effect of a more plausible physiological updating rate, we found that the reconstructed positions still matched the data in excellent accord with the half-angle law for rates as low as 5 to 10 Hz. If we take the standard deviation of Listing’s plane, the expected deviations of the order ξ^2^ are well within the order of magnitude, which have been reported for straight-line and curvilinear pursuit in the position domain [18], [10]–[12], [29].

We have shown that in general the angular orientation of the rotation plane of the total angular eye velocity (equation 3) does not only depend on gaze eccentricity as suggested by the half-angle rule but also on the rotation angle of simultaneous meridian rotations, i.e. rotations in horizontal, vertical or oblique planes. Such rotations affect the tilt of the angular eye velocity rotation plane in two ways: First, it reduces the overall tilt angle compared to that predicted by the half-angle rule (compare tilt profiles in Fig. 6A and 8). The second more subtle effect is that it modulates the tilt angle of the rotation plane of the Donders-Listing angular velocity. During smooth tracking, meridian and Donders-Listing rotations of the eye likely alternate in steps just small enough to avoid catch-up saccades (see Fig. 3A). Since meridian rotations do not affect the torsion of the eye, the underlying ocular rotation remains perfectly in accord with the half-angle law of a Listing-motion in the position domain. During saccades on the other hand the meridian rotation of the eye can be large and thus also alter the orientation of the overall angular velocity rotation plane. Three-dimensional analyses of strongly curved saccades and even single-axis rotation saccades support this prediction [29]–[31]. Vestibular angular velocities may also contribute and change the rotation of the eye in any plane. The particular decomposition of the visually dependent total angular eye velocity shown in equation 3 suggests that the Donders-Listing mechanism still remains in control of fine-tuning eye position during visual-vestibular interactions. Angular eye velocity tilts that do not follow the half-angle rule have been reported during interactions of the translational vestibulo-ocular reflex (known to obey Listing’s law) and the rotational vestibulo-ocular reflexes [32], [33].

Finally, the here presented angular velocity derivation also predicts that the ratio of counter-roll to roll angular velocity should be modulated by the magnitude of the gradient 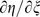 (equation 6). We have earlier shown that during circular tracking this ratio increases proportional to 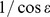 with target-eccentricity ε [18]. During elliptic tracking we found that the angular eye velocity can undershoot this ratio (Fig. 9), indicating that it effectively rolled in the same direction as predicted by equation 6. The successful reconstruction of this effect based on equation 6 corroborates the theoretical assumptions leading to the notion of Donders-Listing rotation operator. Whereas the experimental finding was derived from standard evaluations of the eye position and velocity data, equation 6 was independently derived from the Donders-Listing rotation operator and the thereof derived angular eye velocity.

### Neural Implementation

Our analyses and reconstructions are based on the single assumption of an internal model of the desired motion trajectory across the spherical field of fixations. The appropriate motor commands can be envisaged as a series of position and displacement signals guiding the line of sight along the desired trajectory. Based on these requirements, the superior colliculus appears to be the ideal candidate for this kind of neural processing, given the retinal inputs to this structure and its retinotopic topography [34]–[37] (for a review of the common functional architecture of the pursuit and saccadic system, see [38], [39]). As far as saccades are concerned, neural activity correlated to gradients orthogonal to the classic two-dimensional movement field of collicular cells has not been found [40], [41]. However, the novel features proposed here predict only small displacements in the order of about 5°, distributed along the desired trajectory in a discrete and irregular manner. Theoretically, the coordinates and amplitudes of these signals can be deduced from retinal signals. Structures downstream from the superior colliculus, for example the nucleus reticularis tegmenti pontis and subsequent structures in the ponto-cerebellar pathway may be candidates for this kind of processing [42], [43]. Another interesting aspect concerns the observed cooperation between piecewise smooth eye movements and minute saccades. This cooperative interaction contributed to the modulation of angular eye velocity, which is however hard to separate in terms of position and velocity because of its non-linear nature.

In conclusion, our analysis supports the notion of Helmholtz that Listing’s law serves in a fundamental way to easy visuo-spatial orientation by restricting the visually controlled rotation modes of the eye to two planes at any point of time [2]. Both Donders’ and Listing law follow directly from this restriction. The implementation of these laws does not need much computational power beyond processing of retinal signals in the brainstem and low-level visual centers as perhaps best demonstrated by the chameleon [44].

## Methods

### Ethics Statement

The experimental data used in this study were obtained in the context of a larger project requiring three-dimensional eye movement records in subhuman primates. The animals had a chronic acrylic head implant for restraining the head in the experimental sessions. Three-dimensional eye movements were recorded with the magnetic search coil technique using a dual search coil that was implanted on one or both eyes under the conjunctiva as previously described [18], [45]. All surgery was performed under aseptic conditions and general anesthesia, and postoperative pain treatment was applied for at least three consecutive days. The animals were housed in groups of three to five individuals in a large room cage (18.5 m^2^) with access to day light and under the daily supervision of a clinical veterinarian of the Institute of Laboratory Animals of the University of Zurich. The housing was equipped with climbing devices, shielding and primate toys. Single cages for temporarily separating 1–2 animals from the group were 1 m×1.5 m×1.8 m in width, depth and height (internal dimension 1.5 m^3^). The animals received a rich diet with daily seasonal fruits and fresh vegetables. These behaviorally well trained animals were used over a number of years for several studies. All experimental procedures were in accordance with the recommendations in the Guide for the Care and Use of Laboratory Animals of the US National Institutes of Health. The housing, husbandry and experimental protocols were reviewed, approved and supervised by the Veterinary Office of the Canton of Zurich.

### Experimental Procedures

3D eye movement records were analyzed in a total of six female rhesus monkeys (*Macaca mulatta*), which had been trained to track a small target light moving along straight (two animals) or curvilinear trajectories (four animals) in visual space (for details see [11], [18]). In brief, the animals were seated upright, with the head restrained in a primate chair mounted within an opaque sphere 1.6 m across. A small laser spot (0.35°) was projected onto the inner wall of the surrounding sphere describing linear, circular or elliptic paths on a structured background at a rotation frequency of 0.1 Hz. Linear tracking was tested at eccentricities of 0°, ±10°, and ±15° relative to straight ahead using oscillation amplitudes of 15°. For elliptic tracking, see below. The quality of smooth tracking was controlled with behavioral windows of 1–2° across. All experiments were performed in dimmed light, i.e. with a background illumination inside the opaque sphere, which completely surrounded the animal. Three-dimensional eye positions were measured using the magnetic search coil technique with an Eye Position Meter 3000 (Skalar, Delft, The Netherlands). Three-dimensional (3D) eye position was calibrated as described in Hess et al. (1992) [28], digitized at a sampling rate of 833.33 Hz, and stored on a computer for off-line analysis. To express eye positions as rotation vectors [46], the zero or reference positions were defined to be the eye’s orientations while the monkey fixated a target 0.8 m straight ahead. In four animals, Listing’s plane tilted, respectively, less than 4° vertically and 1° horizontally from the frontal plane. In the other two animals, Listing’s plane tilted vertically −1.2° and −5° and horizontally −3.7° and −3°. We did not correct eye positions for these deviations from primary position (see Discussion).

Vectors in 3D Euclidean space will be denoted by bold characters. Often we refer to equivalent 1- or 2-vectors, which will be denoted by regular characters. Unit vectors will generally be denoted by regular fonts with caret. When referring explicitly to the components, we write vectors for convenience as row vectors within round parentheses, separating the components by commas.

### Encoding 3D Eye Position in Head-fixed Spherical Coordinates

All responses were analyzed cycle per cycle. Saccades, quick phases, and blink artifacts were detected and marked by applying time and amplitude windows to the time derivative of eye acceleration. Cycles with saccades or blink artifacts were eliminated by visual inspection. To facilitate identification of saccadic events in terms of magnitude, duration and peak velocity, eye position traces were rectified by subtracting the sinusoidal modulation determined by least-squares fitting.

Three-dimensional angular eye velocity (**Ω**) was computed with the global formula 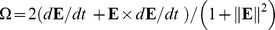 [21]. Thereby, torsional, vertical and horizontal eye position, denoted 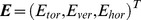 (T stands for transpose) was expressed as rotation vector 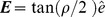 where 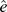 is a unit vector parallel to the axis of rotation, 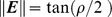 the magnitude and 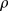 the angle of rotation. We fitted 3D eye position and angular velocity by the method of minimal least squares with a sum of sinusoids up to the 2^nd^ second harmonic of the spatial stimulus frequency. We used the scatter-free sinusoidal-fits to compare the predictions of equation 3 and 6 with experimental data (see Figs. 8 and 9). Using these best-fitting eye position functions, we computed the motion of the unit gaze vector 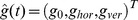 parallel to the line of sight. Note that 
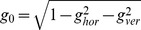
. We call the plane spanned by the line of sight and the direction straight ahead meridian plane. The angular orientation of the unit gaze vector was defined by the angular eccentricity ε relative to straight ahead, ε = ε(t) and the signed meridian angle ψ = ψ(t), subtended by the meridian and sagittal plane, spanned by the head vertical and straight-ahead directions (Figs. 1 to 3). According to the right hand rule, the meridian angle was taken positive in the direction of the curling fingers with the thumb pointing forward, parallel to straight ahead.

### Data Analysis

#### Reconstruction of the listing-motion of the eye based on 3D eye position records

The Listing-motion of the eye was estimated by applying the compound meridian and DL- operator (equation 2) to the unit gaze vector in the spherical field of fixations.

For solving the equations of straight-line tracking in visual space, we assumed the existence of an internal model estimating the target distance and orientation relative to straight ahead in the frontal plane by the two equations 
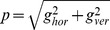
 and 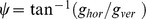 at any instant of time. Thereby, 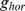 and 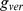 represented the horizontal and vertical component, respectively, of the unit gaze vector in the spherical field of fixation. The associated target eccentricity followed from the relation 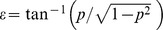. The further procedures were the same as described for the elliptic tracking paradigm in the following paragraphs.

In the elliptic paradigms we first determined the best fit ellipse to the gaze trajectory using the parametric equation 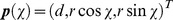, with d, distance between the observer’s eye and the center of the ellipse projected straight-ahead, χ, polar angle measured from the major axis, and 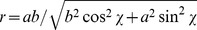, and a, b the semi-major and semi-minor axes. From these fits we obtained the coordinates of the observer’s gaze line when fixating the target: then the eye’s angular eccentricity ε matches the target’s eccentricity relative to the center straight ahead, thus 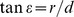 and the meridian angle 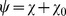. We determined 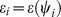 for each sampling point, starting with 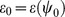, the initial fixation position at tracking onset, and ending with 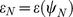 at the end of the cycle. For each gaze position we estimated the underlying incremental rotation angles 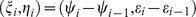 and the associated gradients 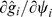 and 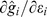. The initial rotation relative to primary position was obtained by applying 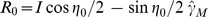 on the unit gaze vector 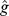 in primary position using 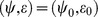 and 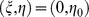. For all subsequent rotations we used 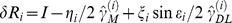 with 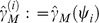 and 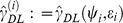. We recursively conjugated the unit gaze vector 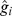 with 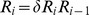, starting with 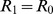 (see equations 1 and 2). All tracking was recorded at a frequency of 0.1 Hz. The total number of samples N of one tracking cycle was N = 8333 corresponding to the sampling rate of 833.33 Hz of the experimental data. Reducing the sampling rate down to about 5 Hz had little effect on the quality of the reconstructed ocular rotation (see example in Fig. 4). The angular eye velocity was reconstructed on the basis of the same series of positions and rotation angles that described the time evolution of the unit gaze vector using equations 3 to 5. Thus, the resulting angular eye velocity represented an average angular eye velocity across all the slow phase segment of a given response cycle similar as the reconstructed eye position.

We also computed the generalized R^2^ values based on the residual sum of squares of the reconstructed and the reduced model consisting of average eye position or angular velocity. Root-mean square errors were computed by evaluating the expression 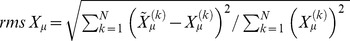, where 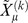 and 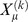 are the k^th^ sample of the µ^th^ component of the experimental and reconstructed angular eye position or velocity, respectively (µ = “tor”, “ver”, or “hor”, N = number of samples).

### Estimation of the Average Gaze Eccentricity per Cycle of Elliptic Tracking

We used elliptic target trajectories with three different eccentricities 
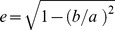
 = 0.66, 0.87 and 0.97 (a = 20°, semi-major axis; b = 15°, 10°, and 5°, semi-minor axes). To estimate the average gaze eccentricity we applied the parametric equation of the elliptic track, centered straight ahead of the subject, 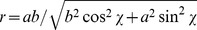 to obtain the average gaze eccentricity from the center per angle, 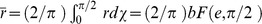 with the elliptic integrals 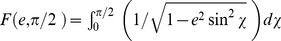 as a function of the elliptic eccentricity e.

### Ratio of Counter-roll to Roll Angular Velocity

We estimated the ratio of counter-roll to roll angular velocity from eye position and angular velocity records as follows: Because the angular velocity in the coronal plane of the eye must be zero, we estimated the angular velocity in eye-fixed coordinates from the recorded angular velocity by setting the torsion component to zero, i.e. we estimate 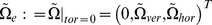. Secondly, we estimated the target-induced angular velocity by 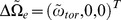 with 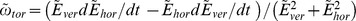 obtained from the recorded eye position 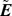 [47]. With these designations the equation for λ reads 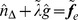, where 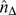 represents the direction of the target-induced angular velocity, 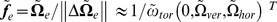 and 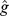 is unit gaze vector computed from 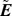. Finally, we chose the parameter λ such that it fulfilled the quadratic equation 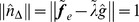 with solutions 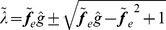. The x-direction of 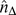 thus is 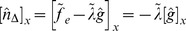 and 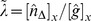, which cancels the target-induced angular velocity: 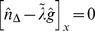 [18]. For statistical comparison, we calculated the generalized R^2^ values by comparing the residual sum of squares obtained from the parametric equation 6 and the associated reduced equation by setting 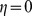 in 6. The normalized root mean square error between the experimentally and parametrically estimated λ was computed based on the formula 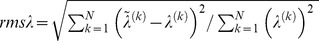 (N = number of samples).

## Supporting Information

Text S1 Clifford algebra and rotations in 3D Euclidean space.
(DOCX)

Text S2Displacements generated by the Donders-Listing operator 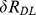 minimize ocular torsion across the oculomotor range.(DOCX)

Text S3 Calculation of the ratio of counter-roll to roll angular velocity.
(DOCX)
