# Supplementary material for: Kinematics of Visually-Guided Eye Movements
Source: PLoS One. 2014 Apr 21;9(4):e95234. doi: 10.1371/journal.pone.0095234 (PMC3994052; doi:10.1371/journal.pone.0095234)
Supplement: Text S1 — Clifford algebra and rotations in 3D Euclidean space. (DOCX) [file pone.0095234.s001.docx]

**Text S1: Clifford algebra and rotations in 3D Euclidean space**

For computing rotations in three-dimensional Euclidean space we used the associated Clifford algebra that is generated by the three numbers$\hat{\gamma}_{1}$, $\hat{\gamma}_{2}$, $\hat{\gamma}_{3}$and unity I with the following properties [1]

$\hat{\gamma}_{j}\hat{\gamma}_{k}+\hat{\gamma}_{k}\hat{\gamma}_{j}=2\delta_{jk}I$ with $\delta_{jk}=1$ if j =k and $\delta_{jk}=0$ if j ≠k

Any linear combination of the$\hat{\gamma}_{i}$’s is a 1-vector, a linear combination of $\hat{\gamma}_{i}\hat{\gamma}_{j}$(i ≠j) is a 2-vector and any multiple of $\hat{\gamma}_{i}\hat{\gamma}_{j}\hat{\gamma}_{k}$(i ≠j ≠k) is a 3-vector. We use the abbreviations $\hat{\gamma}_{ij}:=\hat{\gamma}_{i}\hat{\gamma}_{j}$and$\hat{\gamma}_{ijk}:=\hat{\gamma}_{i}\hat{\gamma}_{j}\hat{\gamma}_{k}$for products of 1-vectors. A general Clifford number u thus can be expressed as $u=aI+b^{i}\hat{\gamma}_{i}+c^{jk}\hat{\gamma}_{jk}+d^{ijk}\hat{\gamma}_{ijk}$, using the Einstein summation convention. The reverse of u, denoted$u^{\dagger}$, is$u^{\dagger}=aI+b^{i}\hat{\gamma}_{i}+c^{jk}\hat{\gamma}_{kj}+d^{ijk}\hat{\gamma}_{kij}$, where the order of all products is reversed, and the scalar component of *u* and $u^{\dagger}$is$\left(u\right)_{0}=\left(u^{\dagger}\right)_{0}=a$. With these definitions at hand, the inner product (or Clifford scalar product) of u and v is, using the dot or bracket notation. The area of a 2-vector thus is $\left|u\right|=\sqrt{\left\langle u,u\right\rangle}=\sqrt{\left(uu^{\dagger}\right)_{0}}$[1, 2].

A rotation of a 1-vector $x$ in the plane$A=\hat{\gamma}_{\alpha\beta}$spanned by the two 1-vectors $\hat{\gamma}_{\alpha}$and $\hat{\gamma}_{\beta}$with $\left|A\right|=1$ through angle ϱ is obtained by the conjugation$x'=R_{A}\left(\rho\right)xR_{A}^{-1}\left(\rho\right)$ with the operator$R_{A}\left(\rho\right)=I\cos(\rho/2)-\sin(\rho/2)\hat{\gamma}_{\alpha\beta}$. The inverse of R_A_ is$R_{A}^{-1}\left(\rho\right)=R_{A}\left(-\rho\right)$. Note that $\hat{\gamma}_{\alpha}$and$\hat{\gamma}_{\beta}$stand for any linear combination of the basis vector $\hat{\gamma}_{i}$under the condition that$\left|\hat{\gamma}_{\alpha}\right|=\left|\hat{\gamma}_{\beta}\right|=1$. In contrast to the Clifford algebra, Hamilton’s approach to represent rotations with generalized complex numbers i, j, k does not make a difference between generators of translations, i.e. vectors, and generators of rotations, i.e. bi-vectors. If $r=r_{1}i+r_{2}j+r_{3}k$ is a pure quaternion or vector and $q=q_{0}+q_{1}i+q_{2}j+q_{3}k$ a unit quaternion,$\left|q\right|=1$ a rotation in Euclidean space is represented by$r'=qrq^{-1}$ [2].

For computational purposes the basis vectors $\hat{\gamma}_{i}$can be represented by the following 4×4 matrices (Dirac matrices)

$I=\left(\begin{matrix} 1 & 0 & 0 & 0 \\ 0 & 1 & 0 & 0 \\ 0 & 0 & 1 & 0 \\ 0 & 0 & 0 & 1 \end{matrix}\right)$, $\hat{\gamma}_{1}=\left(\begin{matrix} 0 & 0 & 0 & 1 \\ 0 & 0 & 1 & 0 \\ 0 & 1 & 0 & 0 \\ 1 & 0 & 0 & 0 \end{matrix}\right)$, $\hat{\gamma}_{2}=\left(\begin{matrix} 0 & 0 & 1 & 0 \\ 0 & 0 & 0 & -1 \\ 1 & 0 & 0 & 0 \\ 0 & -1 & 0 & 0 \end{matrix}\right),\hat{\gamma}_{3}=\left(\begin{matrix} 1 & 0 & 0 & 0 \\ 0 & 1 & 0 & 0 \\ 0 & 0 & -1 & 0 \\ 0 & 0 & 0 & -1 \end{matrix}\right)$

For a more rigorous treatment of the correspondence between vectors, cross vector products and triple products in three-dimensional Euclidean space and 1-vectors, 2-vectors and 3-vectors of the associated Clifford algebra see the pertinent literature, e.g. [2].

1. Snygg J (1997) Clifford Algebra: A computational tool for physicists, New York: Oxford University Press. 335 p.

2. Lounesto P (2001) Clifford Algebras and Spinors, Cambridge: Cambridge University Press. 338 p.
