# Supplementary material for: Kinematics of Visually-Guided Eye Movements
Source: PLoS One. 2014 Apr 21;9(4):e95234. doi: 10.1371/journal.pone.0095234 (PMC3994052; doi:10.1371/journal.pone.0095234)
Supplement: Text S2 — Displacements generated by the Donders-Listing operator minimize ocular torsion across the oculomotor range. (DOCX) [file pone.0095234.s002.docx]

**Text S2: Displacements generated by the Donders-Listing operator** $\delta R_{DL}$ **minimize ocular torsion across the oculomotor range**

We seek the angle $\rho$ as a linear function of the roll angle ξ, that minimizes ocular torsion ξ across the spherical field of fixations under the action of the compound rotation operator$R_{CF}\left(\rho,\xi\right)=R_{C}\left(\rho\right)R_{F}\left(\xi\right)$, where $R_{F}\left(\xi\right)=I\cos\xi/2-\sin\xi/2\hat{\gamma}_{23}$generates rotations of the eye in the frontal plane and $R_{C}\left(\rho\right)=I\cos\rho/2-\sin\rho/2\hat{\gamma}_{\varepsilon\psi}$in the eye’s coronal plane. Defining $\rho=\left(1/k\right)\xi$with k =constant, we have

$$\left.R_{CF}\left(\rho,\xi\right)\right|_{\xi=k\rho}=R_{C}\left(\rho\right)R_{F}\left(k\rho\right)$$

The rate of change with $\rho$is $dR_{CF}/d\rho=\left(dR_{C}/d\rho\right)R_{F}+kR_{C}\left(dR_{F}/d\xi\right)$ with$d\xi/d\rho=k$. Setting$dR_{CF}/d\rho=0$ and expanding the right hand side up to first order in $\rho$ and$\xi$ :

$$I\rho/2-\xi/2\gamma_{\varepsilon\psi}\gamma_{23}+\gamma_{\varepsilon\psi}+k\left(I\xi/2-\rho/2\gamma_{\varepsilon\psi}\gamma_{23}+\gamma_{23}\right)=0$$

Evaluating this relation with$\hat{\gamma}_{\varepsilon\psi}=\hat{\gamma}_{23}\cos\varepsilon-\hat{\gamma}_{31}\sin\varepsilon\sin\psi+\hat{\gamma}_{12}\sin\varepsilon\cos\psi$ , $\hat{\gamma}_{\varepsilon\psi}\hat{\gamma}_{23}=-I\cos\varepsilon-\sin\varepsilon\left(\hat{\gamma}_{12}\sin\psi+\hat{\gamma}_{31}\cos\psi\right)$ and rearranging the terms we obtain:

$AI+B\hat{\gamma}_{23}+C\hat{\gamma}_{31}+D\hat{\gamma}_{12}=0$ , with coefficients:

$A=\rho/2+\xi/2\cos\varepsilon+k\left(\xi/2+\rho/2\cos\varepsilon\right)$, $B=\cos\varepsilon+k$

$C=\sin\varepsilon\left(\xi\cos\psi-\sin\psi\right)$,$D=\sin\varepsilon\left(\xi\sin\psi+\cos\psi\right)$

The conditions for minimal torsion thus are A =0 and B =0, whereas the values of C and D, who determine the relative orientation of the planes $\hat{\gamma}_{31}$ and $\hat{\gamma}_{12}$ do not affect ocular torsion.

We obtain the two conditions $k=-\left(\rho/2+\xi/2\cos\varepsilon\right)/\left(\xi/2+\rho/2\cos\varepsilon\right)$ and$k=-\cos\varepsilon$, independent of the roll position ψ. The first condition says that $k=-1$for$\rho=-\xi$, whereas the second condition says that$k=-\cos\varepsilon\approx-1+\varepsilon^{2}/2$. In the range π/2 > ε >0, the optimal values for k thus increase monotonically. For ε =0, i.e. gaze straight ahead, the compound rotation operator $R_{CF}\left(\rho,\xi\right)$evaluated at $\rho=-\xi$ is the identity, generating zero torsion. For ε >0 the first order approximation $\delta R_{DL}$of $R_{CF}\left(\rho,\xi\right)$ evaluated at $\rho=-\xi$ minimizes torsion up to second order corrections in ε and ξ, independent of roll position ψ.
