# Supplementary material for: Kinematics of Visually-Guided Eye Movements
Source: PLoS One. 2014 Apr 21;9(4):e95234. doi: 10.1371/journal.pone.0095234 (PMC3994052; doi:10.1371/journal.pone.0095234)
Supplement: Text S3 — Calculation of the ratio of counter-roll to roll angular velocity. (DOCX) [file pone.0095234.s003.docx]

**Text S3: Calculation of the ratio of counter-roll to roll angular velocity**

To solve the equation $\left\langle\hat{\gamma}_{\Delta}+\lambda\hat{\gamma}''_{\varepsilon\psi},\hat{\gamma}_{23}\right\rangle=0$for λ, where$\hat{\gamma}_{\Delta}=\Delta\Omega/\left|\Delta\Omega\right|$and$\hat{\gamma}''_{\varepsilon\psi}=\Omega''_{C}/\left|\Omega_{C}\right|$, we first evaluate valuate$\hat{\gamma}_{\Delta}$. We have$\Delta\Omega=\Omega_{M}+\Omega''_{C}$, with$\Omega''_{C}=-d\xi/dtR_{M}^{-1}\hat{\gamma}_{23}R_{M}$and $\Omega_{M}=-d\eta/dt\hat{\gamma}_{M}+2\sin\eta/2d\xi/dt\hat{\gamma}_{\partial M}$. Note that the derivative$d\psi/dt$can be replaced by$d\xi/dt$in smooth steady- state tracking. Expanding the scalar product $\left|\Delta\Omega\right|^{2}=\left\langle\Omega_{M}+\Omega''_{C},\Omega_{M}+\Omega''_{C}\right\rangle$and evaluating each term we obtain:

$\left|\Delta\Omega\right|=d\xi/dtA_{\Delta}$, with$A_{\Delta}=\sqrt{1+2\sin^{2}\eta+4\sin^{2}\eta/2+\left(\partial\eta/\partial\xi\right)^{2}}$

Thus $\hat{\gamma}_{\Delta}$can be expressed in terms of η, ξ and ψ as follows:

$$\hat{\gamma}_{\Delta}=\left(-\partial\eta/\partial\xi\hat{\gamma}_{M}+2\sin\eta/2\hat{\gamma}_{\partial M}-d\xi/dtR_{M}^{-1}\hat{\gamma}_{23}R_{M}\right)/A_{\Delta}$$

For the evaluation of$\lambda=-\left\langle\hat{\gamma}_{\Delta},\hat{\gamma}_{23}\right\rangle/\left\langle\hat{\gamma}''_{\varepsilon\psi},\hat{\gamma}_{23}\right\rangle$, we observe that$\left\langle\hat{\gamma}_{M},\hat{\gamma}_{23}\right\rangle=0$, $\left\langle\hat{\gamma}_{\partial M},\hat{\gamma}_{23}\right\rangle=\sin\eta/2$, $\left\langle R_{M}^{-1}\hat{\gamma}_{23}R_{M},\hat{\gamma}_{23}\right\rangle=-\cos\eta$ and $\left\langle\hat{\gamma}''_{\varepsilon\psi},\hat{\gamma}_{23}\right\rangle=\sum_{i} g_{i}\left\langle R_{M}^{-1}\hat{\gamma}_{jk}R_{M},\hat{\gamma}_{23}\right\rangle$ , noting that $\hat{\gamma}'_{\varepsilon\psi}=R_{F}^{-1}\hat{\gamma}_{\varepsilon\psi}R_{F}=\hat{\gamma}_{\varepsilon\psi}$. Altogether we have

$$\lambda=\left(\cos\eta-2\sin^{2}\eta/2\right)/\left(\left\{g_{1}\cos\eta-\sin\eta\left(g_{2}\sin\psi-g_{3}\cos\psi\right)\right\}A_{\Delta}\right)$$

which can be approximated by $\lambda=1/\left(\hat{g}_{1}\sqrt{1+\left(\partial\eta/\partial\xi\right)^{2}}\right)$for small η.
